# An ENU-induced mutation in Twist1 transactivation domain causes hindlimb polydactyly with complete penetrance and dominant-negatively impairs E2A-dependent transcription

**DOI:** 10.1038/s41598-020-59455-9

**Published:** 2020-02-12

**Authors:** Run-Ze Chen, Xuebo Cheng, Yuexi Tan, Tien-Chien Chang, Hailong Lv, Yichang Jia

**Affiliations:** 1 Tsinghua-Peking Joint Center for Life Sciences, Beijing, China; 2 School of Life Sciences, Tsinghua University, Beijing, China; 3 School of Medicine, Medical Science Building, Room D204, Tsinghua University, Beijing, 100084 China; 4 IDG/McGovern Institute for Brain Research at Tsinghua, Beijing, China

**Keywords:** Bone development, Genetic linkage study

## Abstract

*Twist1* encodes a basic helix-loop-helix transcription factor (TF), which forms homodimer or heterodimer with other TFs, like E2A, to regulate target genes’ expression. Mutations in *TWIST1* are associated with Saethre-Chotzen syndrome (SCS), a rare congenital disorder characterized with osteogenesis abnormalities. However, how dysfunction of TWIST1 leads to SCS is still largely unknown. Here, using an unbiased ENU-induced mutagenesis screening, we identified a novel *Twist1* mutation and the mutant mouse phenocopies some features of SCS in a dominant manner. Physically, our mutation p.F191S lies at the edge of a predicted α-helix in Twist1 transactivation (TA) domain. Adjacent to F191, a consecutive three-residue (AFS) has been hit by 3 human and 2 mouse disease-associated mutations, including ours. Unlike previously reported mouse null and p.S192P alleles that lead to hindlimb polydactyly with incomplete penetrance but a severe craniofacial malformation, our p.F191S causes the polydactyly (84.2% bilateral and 15.8% unilateral) with complete penetrance but a mild craniofacial malformation. Consistent with the higher penetrance, p.F191S has stronger impairment on E2A-dependent transcription than p.S192P. Although human p.A186T and mouse p.S192P disease mutations are adjacent to ours, these three mutations function differently to impair the E2A-dependent transcription. Unlike p.A186T and p.S192S that disturb local protein conformation and unstabilize the mutant proteins, p.F191S keeps the mutant protein stable and its interaction with E2A entire. Therefore, we argue that p.F191S we identified acts in a dominant-negative manner to impair E2A-dependent transcription and to cause the biological consequences. In addition, the mutant mouse we provided here could be an additional and valuable model for better understanding the disease mechanisms underlying SCS caused by TWIST1 dysfunction.

## Introduction

The function of TWIST1 in osteogenesis has been reflected by the identification of the *TWIST1* mutations in Saethre-Chotzen syndrome (SCS), a rare congenital disorder often associated with cone-shaped head, asymmetrical face, hand and foot malformation, and even mental retardation^1–3^. Further studies demonstrated that Twist1 is also involved in epithelial-mesenchymal transition and cell migration during embryonic development and contributes to the invasion of carcinoma cells and tumor metastasis^4–7^.

*Twist1* encodes a basic helix-loop-helix (bHLH) transcription factor and Twist1 DNA-binding capability essential for its functions has been intensively studied previously^8–13^. Twist1 either functions as homodimer or heterodimer with other transcriptional regulators to modulate its DNA binding ability and to determine the ultimate cell fate through different signaling pathways in osteogenesis^14–17^. The dimerization of Twist1 and E2A, another bHLH transcription factor, often leads to activation of target genes^14,17^. However, the dimerization between Twist1 and Hand2 antagonistically determines normal bone morphogenesis, imbalance of which has been implicated in the pathogenesis of polydactyly in rodents^15^. Except for the bHLH DNA binding domain, Twist1 has a C-terminal transactivation (TA) domain, which interacts with other transcriptional factors^18–20^. For example, Twist1 TA domain interacts with Runx2, a member of the Runx family of transcription factors, to inhibit Runx2 transcriptional activity *in vitro* and *in vivo*^18^. However, the detailed mechanisms about how Twist1 regulates osteogenesis and how disease mutations impair Twist1 function to result in the disease are still largely unknown.

Here, using an unbiased ENU-induced mutagenesis screening, we identified a novel Twist1 mutation, p.F191S, responsible for hindlimb polydactyly. Unlike previously reported null and p.S192P alleles that lead to hindlimb polydactyly with incomplete penetrance, our p.F191S leads to the phenotype with complete penetrance (84.2% bilateral and 15.8% unilateral). However, craniofacial malformation is mild in our Twist1-F191S mutants but severe in previously reported *Twist1* insufficient animals. The p.F191S lies at the edge of a predicted α-helix of TA domain, which has been hit by available missense disease-associated mutations found in both human and mouse. Although human p.A186T and mouse p.S192S disease mutations are adjacent to ours, these three mutations function differently to impair E2A-despendent Twist1 transcriptional activity. p.A186T and p.S192S disturb the local protein conformation and unstabilize the mutant proteins, therefore, these two mutations impair E2A-despendent transcription and lead to biological consequences probably through a loss-of-function mechanism. Unlike p.A186T and p.S192S, p.F191S keeps the mutant protein stable and its E2A interaction entire. Therefore, p.F191S may act in a dominant-negative manner to impair E2A-dependent transcription and to cause the phenotypes. In addition, our behavior test does not support that dysfunction of Twist1 impairs learning and memory but social novelty. Therefore, we argue the complexity of SCS disease nature led by different disease-associated mutations, and we hope to provide this additional and valuable disease model to better understand SCS disease mechanisms.

## Results

### Identification of a novel *Twist1* mutation responsible for hindlimb polydactyly

We carried out an ENU-induced mutagenesis screening for inheritable phenotypes in the C57BL/6J background (Supplementary Fig. 1A). A G1 mutant mouse with hindlimb polydactyly was identified and the phenotype was inherited in a dominant manner (Supplementary Fig. 1B,C).

In order to identify the mutation responsible for the polydactyly phenotype, we extracted genomic DNA from a G2 affected mouse and performed the whole exome capture and DNA sequencing^21^. One hundred and sixty mutations were identified by the exome capture and 97% of these mutations are located in the gene regions, including exon, intron, and 5′ and 3′ UTR, reflecting our high exome capture efficiency (Supplementary Fig. 2A). Among these 160 mutations, we did not identify any stop-gain and splicing site mutations, which are often functional mutations induced by ENU^22,23^. Instead, 16 nonsynonymous mutations were identified and all of them were validated by Sanger sequencing in the G2 affected mouse. In order to link the genotype with phenotype, we collected additional 7 affected and 5 unaffected G2 and G3 mice from the mutant family and performed Sanger sequencing for these 16 candidate gene mutations (Table 1). All the sequence results supported that a mutation in *Twist1* (c.572 T > C, p.F191S, NP_035788.1) is responsible for the hindlimb polydactyly phenotype (Supplementary Fig. 2B and Table 1).

**Table 1:** Identification of *Twist1* as mutant candidate gene for polydactyly.

Chr.	Chr. Position	Ref	Mut	Gene	Aff.	Aff.	Aff.	Aff.	Aff.	Aff.	Aff.	Unaff.	Unaff.	Unaff.	Unaff.	Unaff.
1	25588787	C	A	*Adgrb3*	?	?	?	?	?	+	?	−	?	+	?	?
4	42861309	G	A	*Fam205a1*	+	−	?	?	−	+	?	+	−	−	?	?
5	45857492	G	C	*Lcorl*	?	?	?	?	+	+	?	+	+	+	?	?
5	121219276	T	A	*Oas3*	?	?	?	?	+	+	?	+	−	+	?	?
5	141217469	G	C	*Amz1*	?	?	?	?	−	+	?	+	−	−	?	?
6	120423065	T	A	*Il17ra*	−	?	?	?	?	+	?	−	?	−	?	?
9	107233874	T	C	*Hemk1*	−	−	+	−	−	+	?	−	?	+	?	?
9	109048051	T	C	*Fbxw21*	?	?	?	?	−	+	?	+	+	−	?	?
9	119926025	T	G	*Xirp1*	?	?	?	?	?	+	?	+	+	+	?	?
10	23542787	A	G	*Slc18b1*	?	?	?	?	?	+	?	+	+	+	?	?
12	33958549	T	C	*Twist1*	+	+	+	+	+	+	+	−	−	−	−	−
12	58643209	G	A	*Foxa1*	+	−	−	?	?	+	?	−	?	−	?	?
18	37678964	A	G	*Pcdhb22*	−	+	?	?	−	+	?	+	−	−	?	?
6	135152963	C	G	*Hebp1*	?	?	+	?	?	?	?	-	?	−	+	?
7	56131292	G	A	*Herc2*	?	?	?	?	?	+	?	+	?	?	?	?
16	79003433	A	T	*Tmprss15*	?	?	?	?	?	−	?	?	?	?	?	?

Note: 16 nonsynonymous mutant candidates were sequenced in 7 affected and 5 unaffected G2 and G3 mice. All the sequence results consistently support the *Twist1* as the candidate mutant gene for the hindlimb polydactyly (+, carrying this mutation; −, not carrying this mutation; ?, not sequenced).

**Figure 1:**
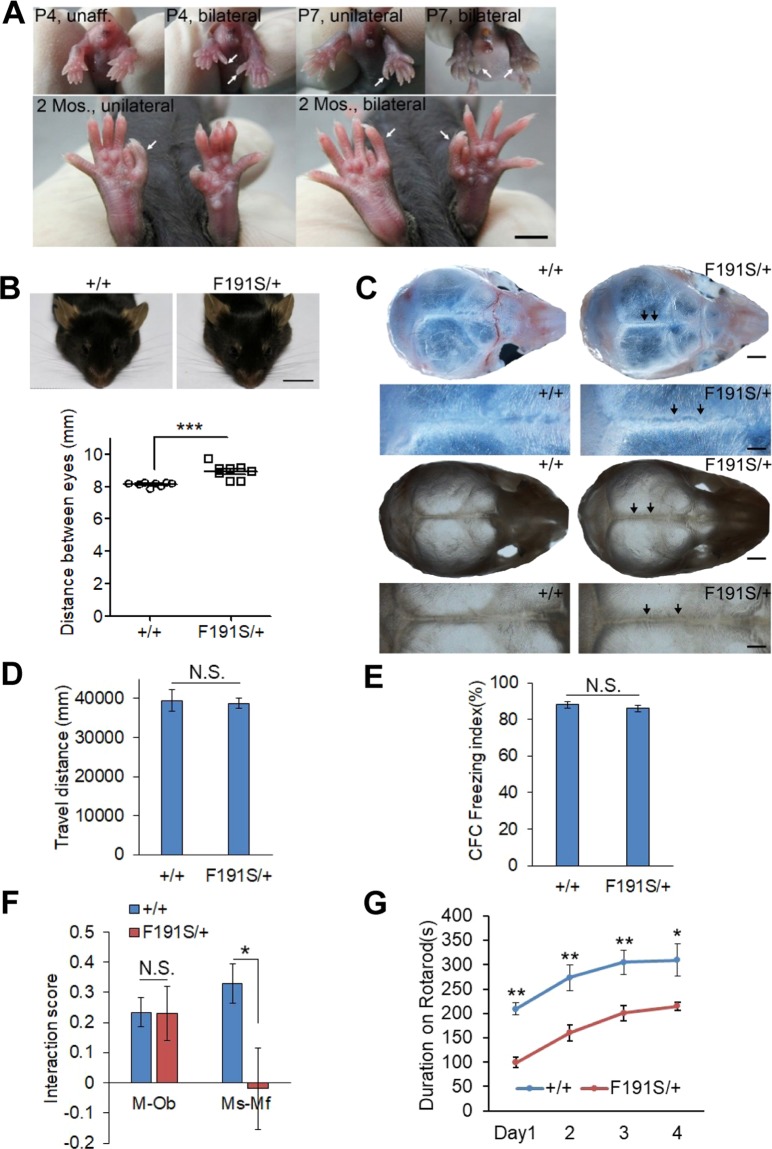
Twist1-F191S mutant mouse carries hindlimb polydactyly and some phenotypic features of the Saethre-Chotzen syndrome. (**A**) The unilateral and bilateral hindlimb polydactyly shown in Twist1-F191S mutant (F191S/+) mice at different ages. A P4 wildtype (+/+) unaffected (unaff.) pup was included. P4 and P7, postnatal day 4 and 7; 2 Mos., 2 month of age. (**B**) The top view of wildtype (+/+) and Twist1-F191S mutant mouse heads (upper, scale bar, 10 mm). The distance between two eyes in wildtype and Twist1-F191S mutant mice was measured (bottom). +/+, n = 8; F191S/+, n = 8. (**C**) Distorted sagittal suture (indicated by arrows) in P10 Twist1-F191S (F191S/+) mutant pups. Scale bar, 1 mm (low mag.), 0.5 mm (high mag.). +/+, n = 6; F191S/+, n = 11. (**D**,**E**) Open field test and contextual fear conditioning (CFC) were carried out in the wildtype (+/+, n = 11) and Twist1-F191S mutant (n = 10–11) mice. (**F**) Three-chamber test for social ability (mouse versus object, M-Ob) and social novelty (stranger versus familiar one, Ms-Mf). Interaction score (M-Ob) = (Time spent with mouse − Time spent with object)/(Time spent with mouse + Time spent with object). Interaction score (Ms-Mf) = (Time spent with stranger − Time spent with acquaintance)/(Time spent with stranger + Time spent with acquaintance). +/+, n = 10; F191S/+, n = 6. (G) Accelerating rotarod test in the wildtype (n = 11) and F191S mutant (n = 11) mice. The values are presented as mean ± SEM. N.S., no statistical significance, *p < 0.05, **p < 0.01, ***p < 0.001 (t-test, SPSS). In B, male, age, 11 months; in D, F and G, male, age, 2 months; in E, male, age, 2.5 months.

In order to confirm that the *Twist1* mutation (p.F191S) is responsible for the hindlimb polydactyly, we crossed the affected mice with the C57BL/6J mice for ten generations (Supplementary Fig. 2C). After that, the *Twist1* mutation is still associated with the phenotype. Meanwhile we disassociated 3 intronic mutations detected by our exome-capture in chromosome 12, the same chromosome *Twist1* located, with the hindlimb polydactyly phenotype (Table 2). However, we failed to obtain mouse homozygous for p.F191S, indicating p.F191S is deleterious to mouse early development. Similar deleterious effect was seen in mouse homozygous for *Twist1* knockout^24^ or for p.S192P^18^. The penetrance of the polydactyly in our mutant family is 100% (Fig. 1A and Supplementary Fig. 2D), and the phenotype appears as early as the first day of birth. The phenotype manifests in both unilateral (16.8%) and bilateral (84.2%) patterns, indicating variable expression in the limb phenotype (Fig. 1A and Supplementary Fig. 2D). Taken together, we identified a novel *Twist1* mutation (p.F191S) responsible for the hindlimb polydactyly with complete penetrance.

**Table 2:** Disassociation of other 3 mutations identified by exsome capture in chromosome 12 with the hindlimb polydactyly phenotype.

Chr	Start	End	Ref	Alt	Mutation location	Gene name	Fraction of DNA-seq	Depth of DNA-seq	Validation	Appearance in affected mouse
12	33958549	33958549	T	C	exonic	Twist1	0.4375	16	Yes	Yes
12	55089706	55089706	T	C	intronic	Srp54a	0.5714	7	Yes	No
12	72775180	72775180	C	T	intronic	Ppm1a	0.5714	7	Yes	No
12	77451898	77451898	T	C	intronic	Fut8	0.4713	244	Yes	No

Note: A G10 affected mouse was used for the disassociation.

### Our Twist1-F191S mutant mouse manifests some features of the Saethre-Chotzen syndrome

Besides the syndactyly, craniosynostosis is another pathological feature shown in Saethre-Chotzen syndrome^1,2^. The craniofacial malformations have been documented in mouse heterozygous for *Twist1* null and for p.S192P allele^18,24^. We observed widely spaced eyes (Fig. 1B) and mild pre-fusion of sagittal suture in our mutant mice with ~54.5% penetrance (Fig. 1C and Supplementary Table 4). However, differently from *Twist1*^−/+^ with high penetrance of pre-fusion of coronal suture (85~95%) and even fusion of lambdoid suture^18^, our mutant mice didn’t display these phenotypes in 11 pups we analyzed (Fig. 1C and Supplementary Table 4).

For some severe cases of Saethre-Chotzen syndrome, patients carry mild to moderate learning disability or mental retardation^3^. However, we did not observe the brain size difference between wildtype and Twist1-F191S mutant mice (Supplementary Fig. 3A). Therefore, we performed behavior assays on our mutant mice. In open field assay, the mutant mice showed normal locomotion (Fig. 1D) and no obvious anxiety-like behavior reflected by normal time spent in the center of the field (Supplementary Fig. 3B). Next, we employed contextual fear conditioning (CFC), a widely-used behavioral paradigm^25^, to examine hippocampus-dependent contextual fear memory in the mutant mice. Compared to the wildtype controls, the mutant mice showed normal sensory response to the electric foot shock and comparable freezing index 24 hours after training (Fig. 1E), indicating our mutant mouse has normal contextual fear memory. To examine whether the mutant mice have social impairment, we employed three chamber test^26^. Similar to wildtype mice, the Twist1-F191S mutant mice spent more time with mouse than with the object, indicating the mutant mouse has normal social ability (Fig. 1F). However, unlike the wildtype mice spending more time with stranger mice than with the familiar ones, the mutant mice didn’t show social preference towards the stranger, indicating that social novelty is impaired by the mutation (Fig. 1F). The social novelty defect seems not due to the impairment of vocal communication, because the numbers of ultrasonic vocalization (USV) between the wildtype and mutant pups after maternal deprivation had no significant difference (Supplementary Fig. 3C).

To examine motor ability, we employed accelerating rotarod paradigm for a consecutive of four-day measurement^27^. On the first day of measurement, the stay time on rotarod was already significantly decreased in the mutant group, indicating a decline in motor ability caused by hindlimb polydactyly in our *Twist1* mutants (Fig. 1G). On the rest days of the measurement, the stay time of the mutant mice was all significantly lower than that of the wildtype (Fig. 1G). However, like the wildtype mice, the stay time of the mutant mice was sharply climbing during the measurement, suggesting the mutant mouse has normal motor learning ability. The motor ability decline seems not caused by body weight difference between the two groups (Supplementary Fig. 3D). Taken together, we concluded that the Twist1-F191S mutant mouse manifests some features of the Saethre-Chotzen syndrome.

### Our p.F191S mutation hits the C-terminal end of Twist1 TA domain

**Figure 2:**
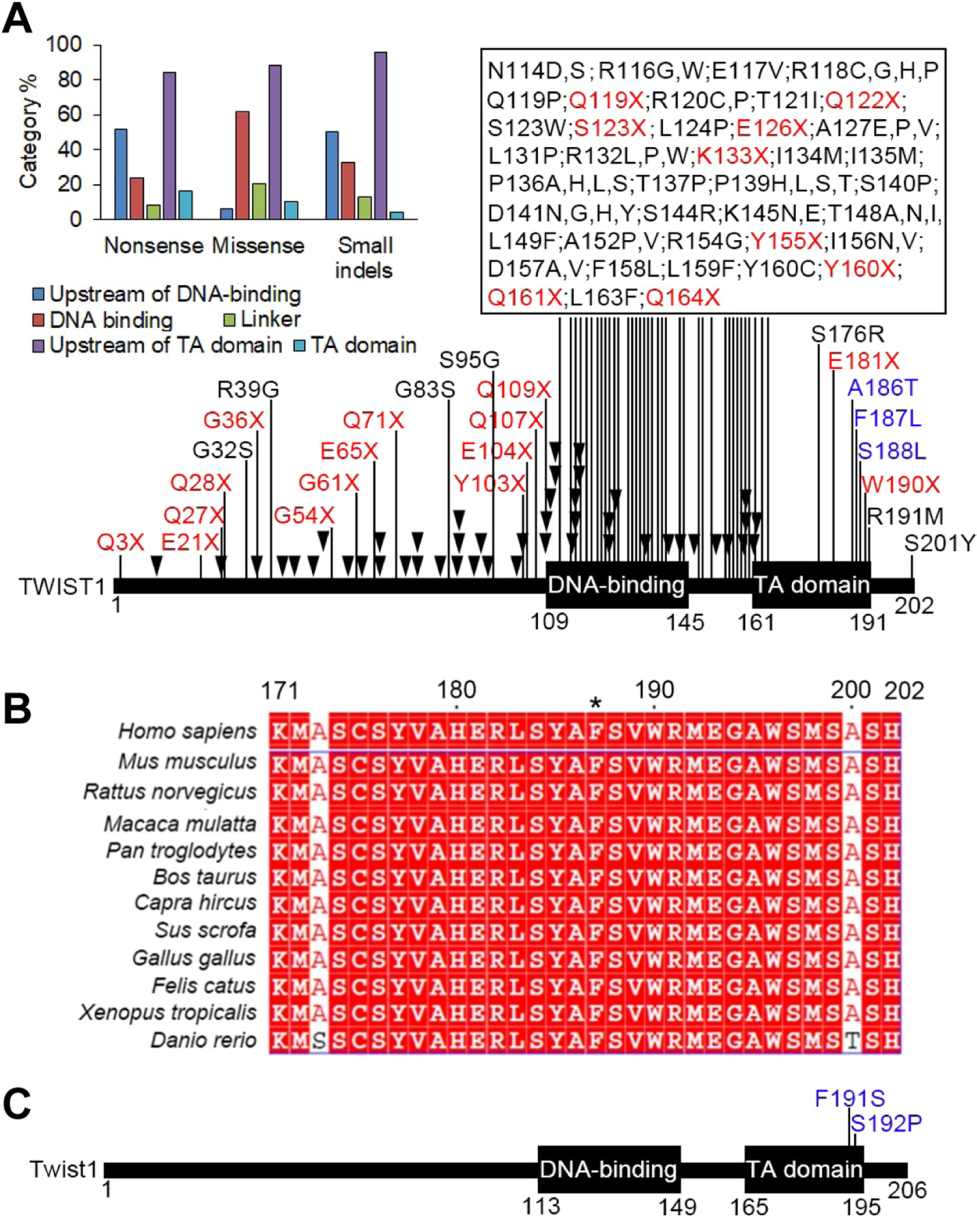
The p.F191S mutation hits C-terminus of Twist1 TA domain in which human disease-associated mutations are clustered. (**A**) Available human TWIST1 pathogenic mutations annotated by HGMD, OMIM, and 1000 Genomes. The disease-associated missense and nonsense mutations were labeled in black and red, respectively. The small indels (insertion and deletion) labeled as black triangles. Three adjacent mutations (p.A186T, p.F187L, and p.S188L labeled in blue, *Homo sapiens*: NP_000465.1) hit the C-terminal end of TA domain. Mouse F191 corresponds to human F187. Inserted: the percentage of missense, nonsense, and small indel mutations occurring in the different parts of TWIST1. (**B**) Protein sequence alignment for the last 29 amino acids of TWIST1 across the species. The amino acids of 186AFS188 (*Homo sapiens*: NP_000465.1) are highly conserved (F187 labelled by asterisk). The NCBI accession numbers: *Mus musculus*: NP_035788.1; *Rattus norvegicus*: NP_445982.1; *Macaca mulatta*: XP_001103003.2; *Pan troglodytes*: NP_001009050.2; *Bos taurus*: NP_001178074.1; *Capra hircus*: XP_005679067.2; *Sus scrofa*: XP_003130240.2; *Gallus gallus*: NP_990070.1; *Felis catus*: XP_003982906.1; *Xenopus tropicalis*: NP_989415.1; *Danio rerio*: NP_571059.1. (**C**) Close to our p.F191S mutation, p.S192P was previously identified in another mouse ENU screening responsible for hindlimb polydactyly (PMID, 15030764). The domain structure of mouse Twist1 annotated by NCBI (NP_035788.1).

The *TWIST1* mutations are associated with Saethre-Chotzen syndrome, a rare congenital disorder often with craniosynostosis, hypertelorism, and syndactyly^1,2^. We collected the disease-associated *TWIST1* mutations from available public databases, including HGMD and OMIM^28–30^. These mutations fall into three categories, including missense/nonsense, small indels (insertion and deletion), and large indels (Fig. 2A and Supplementary Tables 1–3). Among these mutations, the majority of nonsense mutations (84.0%) and small indels (95.7%), which usually generate premature termination codon, occur upstream of TA domain (Fig. 2A). Because the whole coding region of *TWIST1* is encoded by a single exon, the transcripts with these stop-gain mutations most likely would escape the nonsense-mediated decay and generate truncated TWIST1 lacking the TA domain, suggesting the functional importance of this domain as well as TWIST1 DNA binding domain. For missense mutations, the majority of this category hit the DNA binding domain (61.8%), reinforcing that DNA binding is crucial for TWIST1 transcriptional activity. Compared to many missense mutations found in TWIST1 DNA binding domain, we found 6 missense mutations in TA domain (Fig. 2A). However, three (p.A186T, p.F187L, and p.S188L) of them hit three adjacent residues (186AFS188 for human; 190AFS192 for mouse) that are highly conserved across the vertebrates (Fig. 2B), suggesting that these three residues are functionally important for TA domain. Interestingly, another stop-gain mutation (E181X) is upstream of these three resides, which would generate truncated TWIST1 missing the three adjacent residues and the C-terminal end of TA domain. Besides human disease-associated mutations in *TWIST1*, a mouse missense mutation p.S192P (corresponding to human S188) also supports the functional importance of these three residues in Twist1 TA domain (Fig. 2C). The p.S192P was identified in another ENU-induced mutagenesis screening and the mutant mouse displays hindlimb polydactyly similar to what we observed but with incomplete penetrance^18^. Taken together, the p.F191S mutation (corresponding to human F187) we identified hits the C-terminal end of TA domain, in which the disease-associated mutations are clustered.

### The p.F191S lies at the edge of a predicted α-helical structure in the TA domain

Although the 3-D structure of TWIST1 has not been solved, transcription factors with available elucidated 3-D structures that share high sequence similarity with TWIST1 can be served as applicable models for TWIST1 structure prediction. Using this strategy, previous study has modeled the dimerization of TWIST1 DNA binding domain with E2A, together with the E-Box DNA target^31^ (Supplementary Fig. 4). However, so far the structure of the TWIST1 TA domain has not been carefully predicted.

**Figure 3:**
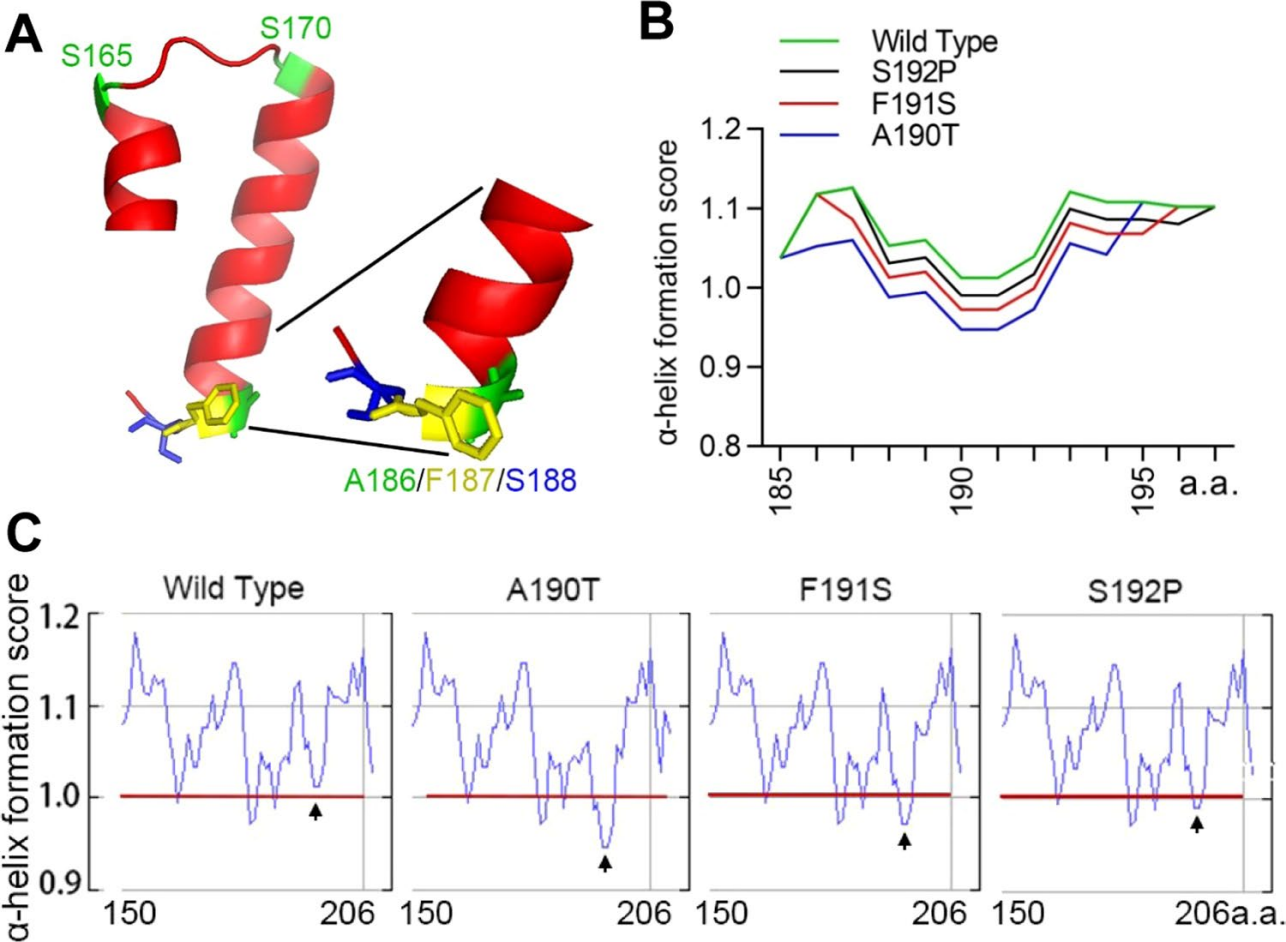
The p.F191S mutation at the edge of a predicted α-helix has mild alteration to the α-helix formation. (**A**) Three dimensional structure of human TA domain (161–191, *Homo sapiens*: NP_000465.1), which adopts a helix-loop-helix structure. The A186 is inside of the predicted α-helical structure and F187 lies at edge of the α-helix. In contrast, S188 is outside of the α-helical structure. The magnified details of A186-S188 location relevant to the α-helix were inserted. A small loop (S165–S170) links the α-helix with DNA binding domain. The structure is predicted by SWISS-MODEL and arranged by PyMOL. (**B**) Prediction of the α-helix formation score for the three adjacent mutations. The mouse Twist1 (185–197) fragments, including wildtype and mutant forms (p.A190T, p.F191S, and p.S192P), were applied for the analysis. (**C**) The raw graph (mouse Twist1 150–206a.a.) of the α-helix formation score predicted by the online program Chou & Fasman analysis (http://web.expasy.org/protscale/). The score change of the mutations was pointed by arrow.

Using an online program^32–35^, we predicted that the TA domain (human 161–191) adopts a helix-loop-helix conformation and F187 (F191 for mouse) lies at the edge of the C-terminal α-helix (Fig. 3A and Supplementary Fig. 4). This α-helix probably connects to the DNA binding domain through a short loop (S165-S170 human), which makes it flexible for conformational change.

Next, we asked whether p.F191S mutation has any influence on the C-terminal α-helix formation. Using Chou & Fasman secondary structure prediction method^36^, we examined the α-helix formation score for Twist1 wildtype and mutants (p.A190T, p.F191S, and p.S192P corresponding to mouse Twist1) (Fig. 3B,C). The human disease mutation p.A186T (mouse A190), which is located more proximal towards the α-helix, has the strongest effect on disturbing the α-helix formation among these three mutations we examined. The mouse disease mutation p.S192P has the mildest effect on the α-helix formation, though the substitution from serine to proline, a rigid amino acid, leads to severe distortion of local structure (Fig. 3B,C). The fact that the S192 lies outside of the α-helix may explain the mild effect of p.S192P on the α-helix formation (Fig. 3A and Supplementary Fig. 4). Our p.F191S mutation appears intermediate effect on the α-helical structure. Therefore, our structure prediction suggested that the p.F191S is located at the edge of the C-terminal α-helix and had mild effect on α-helical structure formation.

### The p.F191S mutation impairs the E2A/Twist1 transcriptional activity but not E2A/Twist1 interaction

**Figure 4:**
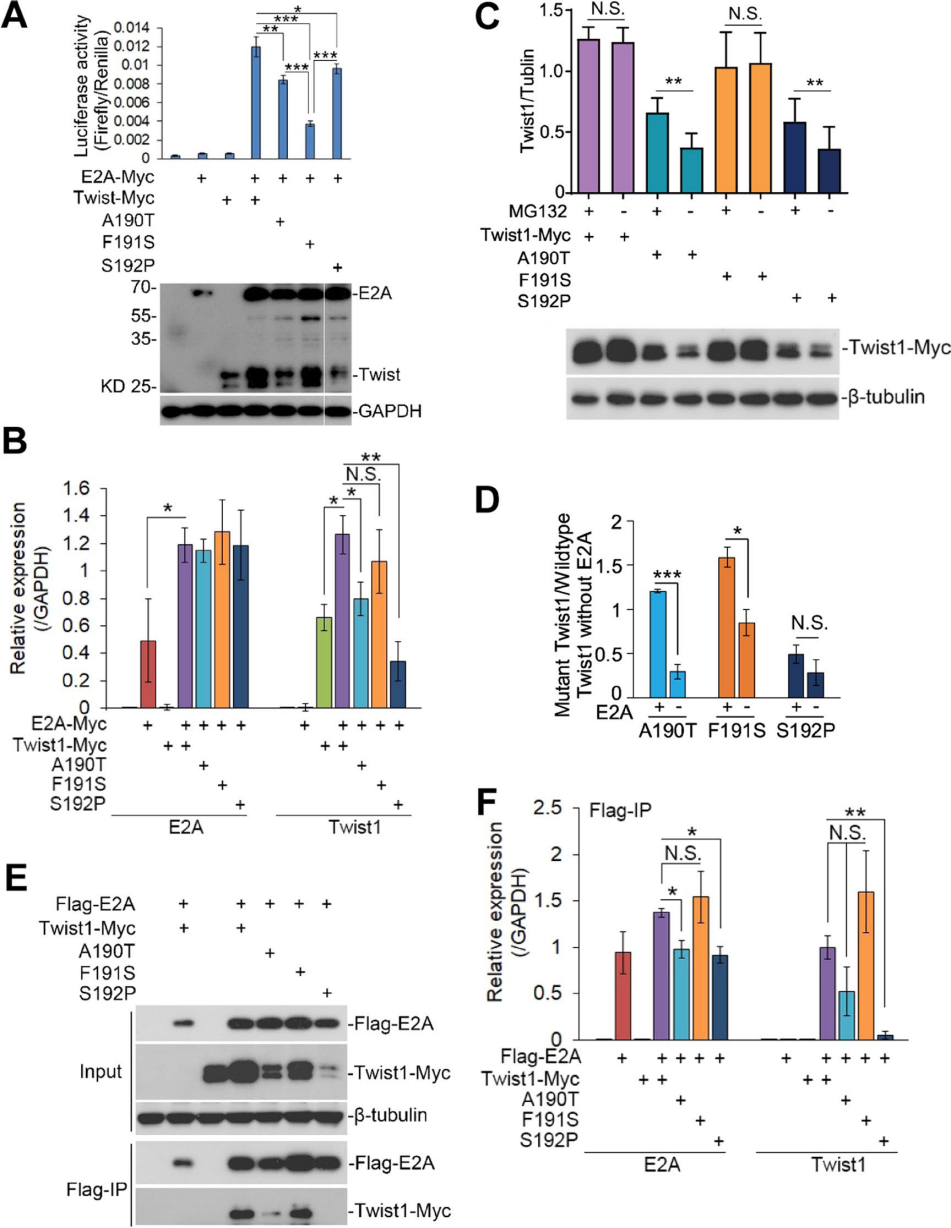
The adjacent p.A190T, p.F191S, and p.S192P mutations affect E2A-dependent Twist1 transcription through different mechanisms. (**A**) The transcriptional activity of wildtype and three Twist1 mutations (p.A190T, p.F191S, p.S192P) measured by E-Box luciferase reporter assay. The E2A and E-Box-Luc were co-transfected with wildtype or mutant Twist1. Lower panel, representative western blot of the cell lysis used for luciferase assay in the upper panel. Myc antibody was used to detect protein expression of both E2A and Twist1. GAPDH served as loading control. The last lanes of Myc and GAPDH were cropped from original blots, which were included in Supplementary Fig. 6. (**B**) Quantitative analysis of E2A and Twist1 expression level shown in (A) by ImageJ. (**C**) The three mutant Twist1 were expressed in presence or absence of a proteasome inhibitor MG132. Here the E2A-expressing plasmids were not co-transfected. (**D**) The relative expression of mutant Twist1 in presence (**A**) and absence (**C**) of E2A. The expression of the mutant Twist1 was normalized to that of wildtype Twist1 in absence of E2A expression. In (**A**), normalized to lane 3; in (**C**), normalized to lane 2. (**E**) Co-immunoprecipitation was carried out to test the protein-protein interaction between E2A and wildtype or mutant Twist1. (**F**) Quantitative analysis of data shown in (**E**) by ImageJ. In (**A–D**,**F**), the value are presented as mean ± SEM (n = 3). *p < 0.05, **p < 0.01, ***p < 0.001 (ANOVA or t-test, SPSS).

Previously, the E2A-dependent Twist1 transcriptional activity has been characterized through both biochemical assay and computational analysis^19,31^. In order to know whether our mutation influences the transcriptional activity, we employed the E-box promoter assay^31,37^. Consistent with previous studies, the luciferase activities were significantly increased in the presence of both E2A and Twist1, but not E2A or Twist1 alone, nor mock control (Fig. 4A). The luciferase activities were greatly decreased when we transfected Twist1-A190T or Twist1-S192P together with E2A, suggesting that these two mutations caused the disease through impairment of Twist1-dependent transcription. Strikingly, our p.F191S mutation had even stronger impairment on the luciferase activity than p.A190T and p.S192P (Fig. 4A), consistent with the higher penetrance of hindlimb polydactyly in Twist1-F191S mutant mice than that in Twist1-S192P.

As hetero-dimer, E2A and Twist1 stabilize each other^38^ (Fig. 4A,B). In contrast, the expression level of Twist1-A190T or Twist1-S192P was significantly decreased even in the presence of E2A, suggesting that these two mutant proteins either were themselves unstable or affected E2A/Twist1 complex formation (Fig. 4A,B). Interestingly, unlike the Twist1-A190T and Twist1-S192P, the expression level of Twist1-F191S was comparable to wildtype Twist1 (Fig. 4A,B). To test whether the three mutations affect the protein stability, we expressed wildtype and three mutants, respectively, in the absence of E2A expression (Fig. 4C). Compared to the expression level of wildtype Twist1 and Twist1-F191S, the expression level of Twist1-A190T or Twist1-S192P was much less, which could be partially restored by MG132, a proteasome inhibitor, suggesting that the two mutant proteins themselves are unstable but not Twist1-F191S (Fig. 4C).

To test whether the three mutations affect interaction between E2A and Twist1, we calculated the relative expression of the three mutant proteins in presence or absence of E2A, since the E2A/Twist1 interaction enable to stabilize each other (Fig. 4A,B). The presence of E2A significantly stabilized the expression of Twist1-A190T and Twist1-F191S but not Twist1-S192P, suggesting that p.A190T and p.F191S did not but p.S192P did affect the interaction (Fig. 4D). This notion was further supported by the experiments in which Twist1-A190T and Twist1-F191S but not Twist1-S192P was co-immunoprecipitated by tagged E2A (Fig. 4E,F). Therefore, we concluded that the impairment of p.F191S on E2A-dependent Twist1 transcriptional activity does not result from impairing Twist1-F191S protein stability nor disrupting the E2A/Twist1 interaction, which differs from the other two adjacent disease mutations, human p.A186T and mouse p.S192P.

## Discussion

### Identification of a novel Twist1 mutation responsible for hindlimb polydactyly

In ENU-induced mutagenesis, mutations that generate stop codon (nonsense), disrupt splicing site, or change amino acid (missense) are most likely disease-causing^22,23^. Among our 160 mutations identified by our exome-sequencing, no stop-gain and splicing site mutation was found. Instead, we identified 16 nonsynonymous mutations and disassociated the other 15 mutations, except for Twist1 p.F191S, with the phenotype (Table 1). We also disassociated 3 intronic mutations in chromosome 12, the same chromosome that *Twist1* is located, with the phenotype (Table 2). Similar to early developmental lethality observed in homozygous mutant mice for *Twist1* knockout^24^ and Twist1 S192P^18^, we failed to produce mutant pup homozygous for p.F191S mutation. More importantly, after ten-generation backcrossed to wildtype C57BL/6J, our p.F191S mutation was still associated with hindlimb polydactyly with 100% penetrance (Supplementary Fig. 2C,D). Lastly, like p.S192P, p.F191S significantly affected E2A-dependent Twist1 transcription activity *in vitro* (Fig. 4A). However, we cannot rule out the possibility that additional unidentified protein coding or non-coding mutations induced by ENU might be in linkage disequilibrium with Twist1 F191S.

### Twist1-F191S mutant mouse manifests hindlimb polydactyly with complete penetrance, craniofacial abnormalities, and social novelty deficit

Previous studies showed that mouse heterozygous for *Twist1* knockout and p.S192P mutation displayed hindlimb polydactyly with 25% and 82% penetrance, respectively^24,39^. Our Twist1-F191S mutant mouse showed 100% penetrance with variable expression in the limb of unilateral (15.8%) and bilateral (84.2%) polydactyly (Fig. 1A and Supplementary Fig. 2D). In agreement with higher phenotypic penetrance *in vivo*, the p.F191S mutation had stronger impairment on E2A-dependent transcriptional activity than p.S192P *in vitro* (Fig. 4A).

Patients with *TWIST1* mutations often carry hand and foot abnormalities, including brachydactyly, syndactyly, and clinodactyly, but not polydactyly^3^. However, mouse carrying null, p.S192P, and our p.F191S mutant alleles consistently display hindlimb polydactyly^18,24^. Obviously, the shapes of limbs between human and mouse are different, which must be finely regulated by many genes and signaling cascades. Some of them have been carefully studied, including *Twist1*, *Hand2*, *Runx2*, *Gli3*, and *Shh* pathway^15,18,40^. The phenotypic difference between human and mouse would be due to slight functional difference between human TWIST1 and mouse Twist1 in limb patterning regulation. In addition, the penetrance of *Twist1* mutations varies between different strains and even depends on epigenetic status^24,41^. Therefore, the phenotypic difference between human and mouse could be explained by different genetic environment and epigenetic status between the two species.

Regarding the craniofacial abnormalities, although we did see widely spaced eyes and pre-fusion of sagittal suture in our mutant pups, the craniofacial malformation is mild (Fig. 1B,C and Supplementary Table 4), compared to *Twist1*^−/+^ ^18^. In addition, we did not see pre-fusion of coronal and lambdoid sutures (Fig. 1C and Supplementary Table 4), which were manifested with high penetrance in the *Twist1*^−/+^ pups^18^. This discrepancy fits our model that p.F191S mutation leads to the phenotypes not through a plain loss-of-function mechanism but in a dominant-negative manner (Supplementary Fig. 5).

In addition to hindlimb polydactyly and mild craniofacial abnormalities, our mutant mouse shows social novelty but not social ability defect (Fig. 1F). Our behavior results do not support the notion that dysfunction of Twist1 leads to learning and memory deficit (Fig. 1E), which is consistent with the previous report that higher mental retardation frequency is shown in patients with a large deletion occupying *TWIST1* and nearby genes than in those with a only *TWIST1* mutation^3^. Therefore, our Twist1-F191S mutant mouse could be an additional and valuable model to study disease mechanisms underlying Saethre-Chotzen syndrome caused by dysfunction of TWIST1.

### Three mutations in the three adjacent residues (186AFS188) lead to disease through different mechanisms

In this study, we identified a novel *Twist1* mutation (p.F191S) responsible for the phenotypes. By collecting available human and mouse disease-associated *Twist1* mutations, together with ours, we found 8 missense mutations in TA domain (Fig. 2A,C). Strikingly, five of them hit three adjacent residues (186AFS188 for human; 190AFS192 for mouse), which are located in the distal end of a predicated α-helix structure (Fig. 3A). However, our data suggests that the three disease mutations we examined, p.A190T, p.F191S, and p.S192P, have different influence on α-helix formation (Fig. 3B,C). p.A190T has the severest effect on the α-helix formation, which may explain why the Twist1-A190T alone is unstable (Fig. 4C,D). Because S192 is outside of α-helix, p.S192P has the mildest effect on the α-helix formation (Fig. 3). However, the substitution from serine to proline, a rigid amino acid, strongly changes the local structure, which may explain why the expression of Twist1-S192P is unstable (Fig. 4C,D). So, p.A190T and p.S192P probably disrupt local structure of Twist1 and lead to mutant Twist1 protein degradation mediated by proteasome.

Different from Twist1-A190T and Twist1-S192P, expression level of Twist1-F191S is as stable as wildtype (Fig. 4C), supported by the mild alternation on α-helix formation by p.F191S (Fig. 3). In addition, Twist1-F191S does not impair the interaction with E2A, but significantly reduces the E2A-dependent transcription (Fig. 4). We hypothesize that the side chain of F191 protrudes at the edge of α-helix (Fig. 3A), which may contribute to an interface for recruitment of other transcriptional cofactors. The hydrophobic F to polar S substitution may destroy the protein-protein interaction interface and affects the recruitment of transcriptional cofactors (Supplementary Fig. 5). Therefore, p.A190T and p.S192P mutations cause the phenotype through a loss-of-function mechanism, while, p.F191S causes the phenotype in a dominant-negative manner without affecting Twist1 expression (Supplementary Fig. 5).

### Dysregulation of dimerization homeostasis contributes to abnormal osteogenesis

The homeostasis between association and disassociation of bHLH transcription factor(s) that forms homodimer or heterodimer to bind DNA is crucial for the regulation of downstream genes^42^. This homeostasis should be precisely modulated and disruption of the balance may lead to abnormal signaling pathways and biological consequences. Previous studies revealed the ratio between E2A-Twist1 (E/T) heterodimer and Twist1-Twist1 (T/T) homodimer that presents in the gene regulatory elements is critical for target gene expression, which is altered in *Twist1*^−/+^ mutant mouse^14,43^. The instability of Twist1-A190T and Twist1-S192P is likely to result in the imbalance, as shown in *Twist1*^−/+^ mutant mouse. In contrast, Twist1-F191S stabilizes the E/T heterodimer (Fig. 4), which negatively regulates FGF signaling^14,17^ and may explain mild craniofacial malformations shown in our mutant mice (Fig. 1C).

Previous study has shown that phosphorylation of T125 and S127 inside of Twist1 DNA binding domain affects Twist1/Hand2 heterodimer interaction^15^. Due to our p.F191S in TA domain that is far from T125 and S127, we speculate that p.F191S may have low chance to affect the dimerization between Twist1 and Hand2. Instead, Twist1 interacts with Runx2 through its TA domain and the interaction prevents Runx2 DNA binding^18^. In addition, heterozygous knockout for *Runx2* rescues the phenotypes caused by *Twist1*^−/+^ ^18^. Therefore, our p.F191S may impair interaction between Twist1 and Runx2 and result in hindlimb polydactyly. It will be intriguing to test this hypothesis in the further study.

## Materials and Methods

### ENU induced mutagenesis and mouse behavior assay

Dominant ENU induced mutagenesis screening was performed according to previous reports^44^. Briefly, C57BL/6J male mice (8–9 week-old) received a dose of ENU (80–110 mg/kg) weekly for a consecutive of 3 weeks by intraperitoneal administration. After infertility test, the ENU-injected males (G0) were mated with C57BL/6J females to generate G1 offspring. The G1 mice with desired phenotypes were crossed to C57BL/6J mice to test inheritance of the phenotypes and establish the family. Our laboratory animal facility has been accredited by AAALAC (Association for Assessment and Accreditation of Laboratory Animal Care International). All mice in our study were maintained with a 12:12 hour light-dark cycle under controlled temperature of 20 °C. All animal handling procedures were conducted at the laboratory animal facility of Tsinghua University. All animal behavior assays were carried out according to the guidelines of the Animal Welfare Act and NIH policies, and were approved by the IACUC (Institutional Animal Care and Use Committee) of Tsinghua University.

For open field test, a square box (50 × 50 × 50 cm) and TopScan video tracking system (CleverSys USA) were employed. Mice were placed in the center of the field and video-tracked for 10 minutes.

The rotarod performance was measured by an automated system (Med Associates, Inc). In brief, the animal was placed on an accelerating spindle (4–40 rpm, 5-minute/trail, 10-minute break/trail, 3 consecutive trials/day) for four days. The fall time from the spindle was auto-calculated by the system when the mouse fell off the spindle within the 5-minute interval. The stay time was calculated by subtraction of the fall time from the 5 minutes, and the mean value of the stay time from 3 consecutive trials per day was used for statistical analysis.

For social behavior test, we followed protocol described in a previous report^26^. A rectangular box (60 length × 40 width × 25 height, cm) consisted of three chambers (20 × 40 × 25 cm) side by side. Two doors (10 × 10 cm) connect the three chambers and allow the animal freely move in the three compartments. After 10-minute habituation, the mouse stay time in two side chambers was calculated. For social ability, a caged mouse was placed in one side and an empty cage (object) was placed in the other side. The social ability interaction score (M-Ob) = (Time spent with mouse − Time spent with object)/(Time spent with mouse + Time spent with object). For social novelty, a caged stranger was placed in one side and a caged acquaintance was placed in the other side. The social novelty interaction score (Ms-Mf) = (Time spent with stranger − Time spent with acquaintance)/(Time spent with stranger + Time spent with acquaintance).

For ultrasonic vocalization (USV) measurement, pup was separated from its mom at postnatal day 7 and placed in a USV measurement machine (Med Associates, Inc.). We recorded 20–100 kHz bands with a cutoff of 40 db. Times of USV were calculated by counting the numbers of USV peaks in 5 minutes.

### Exome capture, DNA sequencing, and mutation identification

Mouse genomic DNA from an affected and an unaffected mouse was extracted by TIANamp genomic DNA extraction kit (TIANGEN). The genomic DNA was sheared into 200–500 bp fragments. The exome captured sequencing library was constructed according to the manual (SeqCap EZ Library SR, Roche)^23^. In brief, the fragmented DNA was size selected, ligated with adaptor, and amplified. The resulting DNA fragments were captured by SeqCap EZ probe pool. After wash, the captured DNA was recovered and amplified again. The quality of the enriched exome DNA was determined by 2100 bioanalyzer (Agilent). The libraries were sequenced through HiSeq. 2500 platform. The DNA sequencing results were subjected to bioinformatics analysis to recover the potential variants caused by ENU. The reads were aligned to mouse reference genome (mm10) using HISAT v2.0.2^45^. The generated BAM files were sorted and duplicated reads were removed by Picard v1.139 (http://broadinstitute.github.io/picard). More than 96% of the sequencing data were located to gene body. In this screening, a variant was considered candidate only when it met three criteria: (a) the number of supporting reads > 4, (b) the number of supporting reads/the depth of this locus >0.3 and <0.8, whereas there was no such variant in the unaffected sample, and (c) according to the annotation by ANNOVAR^46^, functional mutations were extracted.

### Plasmid construction

Mouse *E2A* and *Twist1* coding sequences with ATG were amplified with primers as follows. E2A-F: CGGGATCCATGATGAACCAGTCTCAGAGAATGGCACCCG; E2A-R: CCCAAGCTTCAGGTGCCCGGCTGGGTTGTGGGCCTC; Twist-F: CGGGATCCATGATGCAGGACGTGTCCAGCTCG: Twist-R: CCCAAGCTTGTGGGACGCGGACATGGACCAG. The PCR products of *E2A* or *Twist1* were cloned into pCMV-3Tag-4 vector (Agilent Technologies) through BamHI and HindIII. For the construction of E-Box-luc plasmid, two single strand DNA oligo were synthesized and annealed. E-Box-F: CGAATTCAACATGTGTGATTCGCATGTGTGGACCGGATCCGTGATGCAACATATGGCGGCCATATACTCGAG; E-Box-R: AGCTTCTCGAGTATATGGCCGCCATATGTTGCATCACGGATCCGGTCCACACATGCGAATCACACATGTTGAATTCGGTAC. The *E-Box* DNA was cloned into pGL4.12 [Luc2CP] (Promega). Site-directed mutagenesis was employed to generate *Twist1* mutants^47^. The primers for mutagenesis of *Twist1*-A190T, -F191S and -S192P are A190T-F: GCTCAGCTACACCTTCTCCGTCTGGAGGATGGAGGGGGCCTG; A190T-R: ACGGAGAAGGTGTAGCTGAGCCGCTCGTGGGCCACATAGC; F191S-F: AGCTACGCCTCCTCCGTCTGGAGGATGGAGGGGGCCTGGTC; F191F-R: TCCAGACGGAGGAGGCGTAGCTGAGCCGCTCGTGGGCCACATAG; S192P-F: AGCTACGCCTTCCCAGTCTGGAGGATGGAGGGGGCCTGGTCCATG; S192P-R: ATCCTCCAGACTGGGAAGGCGTAGCTGAGCCGCTCGTGGGCCAC. All the constructs were sequenced before transfection.

### Cell culture, co-immunoprecipitation, and protein degradation assay

HEK293T cells were maintained in DMEM culture medium supplemented with 10% (v/v) FBS, 50 U/mL penicillin, and 50 μg/mL streptomycin in a humidified incubator with 5% CO_2_ at 37 °C. Cells were lysed with lysis buffer (20 mM Tris-HCl, pH 7.5, 1 mM EDTA, 150 mM NaCl, 0.5% NP-40) supplemented with protease inhibitor cocktail and PMSF. The cell lysis was centrifuged at 10,000 × g for ten minutes. One tenth the volume of the supernatants was pipetted as input samples. The remaining supernatants were incubated with M2 Magnetic Beads (M8823, Sigma) overnight by the standard protocol. For protein degradation assay, 10 μM MG132 was added to the culture medium just before plasmid transfection and then incubated for 16–24 hours.

### Dual-luciferase reporter assay system

Luciferase activity was measured by the Dual Luciferase Reporter Assay (Promega, E1960). Briefly, the HEK293T cells were co-transfected with pCMV-3Tag-4-E2A, pCMV-3Tag-4-Twist1 (or Twist1 mutants), pGL4.12-E-Box (firefly luciferase) and pGL4.74 (renilla luciferase) by Lipofectamine 2000 or were transfected with the indicated plasmids. The pGL4.74 was used as an internal control. The pCMV-3Tag-4 empty vector was used as MOCK control. Twenty-four hours after transfection, the cells were harvested and lysed by Promega cell lysis buffer. The E2A dependent Twist1 transcriptional activity was determined by ratio of firefly luciferase to renilla luciferase.

### Western blotting

The denatured cell lysates were subjected to 10% SDS-polyacrylamide (PAGE) gel and proteins were transferred to polyvinyl difluoride (PVDF) membrane (Hybond, Amersham Biosciences). The protein-bound membrane was blocked with blocking buffer at room temperature for 1 hour. Primary antibodies (Myc antibody, M20002L, Abmart; GAPDH antibody, mAb #2118, Cell Signaling; Flag antibody, M20008, Abmart; β-tubulin antibody, M20005, Abmart) were applied to PVDF membrane at 4 °C overnight with gentle shaking. After wash with PBST, the membrane was incubated with appropriate secondary antibodies (GE Healthcare). ImageJ was used to quantify the protein abundance and GAPDH was used as loading control.

### Protein alignment and structure analysis

Alignment of Twist1 protein from different species was done by submitting the accession numbers (*Mus musculus*: NP_035788.1, *Homo sapiens*: NP_000465.1, *Rattus norvegicus*: NP_445982.1, *Macaca mulatta*: XP_001103003.2, *Pan troglodytes*: NP_001009050.2, *Bos taurus*: NP_001178074.1, *Capra hircus*: XP_005679067.2, *Sus scrofa*: XP_003130240.2, *Gallus gallus*: NP_990070.1, *Felis catus*: XP_003982906.1, *Xenopus tropicalis*: NP_989415.1, *Danio rerio*: NP_571059.1) to NCBI cobalt constraint-based multiple protein alignment online tool (http://www.ncbi.nlm.nih.gov/tools/cobalt/cobalt.cgi?link_loc=BlastHomeAd) and further submitting to espript 3.0 (http://espript.ibcp.fr/ESPript/cgi-bin/ESPript.cgi)^48^. Human Twist1 3-D structure was predicted by SWISS-MODEL online tool (http://swissmodel.expasy.org/)^32–34,49,50^. The 3-D structure of Twist1 was calculated and arranged by the program PyMOL. For secondary structure prediction of the TA domain, we used online program of Chou & Fasman analysis (http://web.expasy.org/protscale/) to calculate the α-helix formation score^36^.

## Supplementary information

Supplementary Fig 1–6.

Supplementary Table 1.

Supplementary Table 2.

Supplementary Table 3.

Supplementary Table 4.
