# Supplementary material for: An ENU-induced mutation in Twist1 transactivation domain causes hindlimb polydactyly with complete penetrance and dominant-negatively impairs E2A-dependent transcription
Source: Sci Rep. 2020 Feb 12;10:2501. doi: 10.1038/s41598-020-59455-9 (PMC7016005; doi:10.1038/s41598-020-59455-9)
Supplement: Supplementary file 1 — Supplementary Fig 1–6. [file 41598_2020_59455_MOESM1_ESM.pdf]

An ENU-induced mutation in Twist1 transactivation domain causes hindlimb polydactyly with complete penetrance and dominant-negatively impairs E2A-dependent transcription.

Run-Ze Chen[1,2,3,4,5], Xuebo Cheng[1,3,4,5], Yuexi Tan[1,2,3,4], Tien-Chien Chang[3], Hailong Lv[1,2,3,4], Yichang Jia[1,3,4, #]

1 Tsinghua-Peking Joint Center for Life Sciences.
2 School of Life Sciences, Tsinghua University
3 School of Medicine, Medical Science Building, Room D204, Tsinghua University, Beijing, China, 100084.
4 IDG/McGovern Institute for Brain Research at Tsinghua.
5 These authors contributed equally to this work.

# Corresponding author
Please address correspondence to:
Yichang Jia, Ph.D.
School of Medicine, Medical Science Building, Room D204, Tsinghua University, Beijing, 100084, P. R. China
Tel: 86-10-62781045
Email: yichangjia@tsinghua.edu.cn

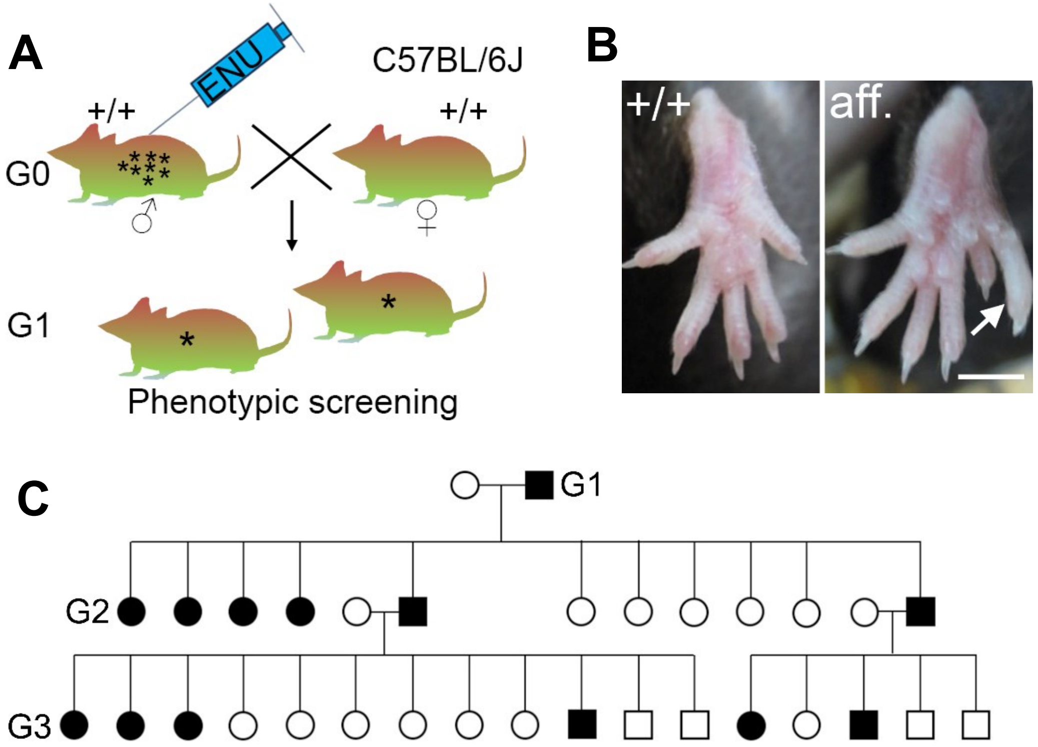

Supplementary Fig. 1. ENU-induced mutagenesis screening for inheritable phenotypes. (A) An ENU-induced mutagenesis G1 screening for inheritable phenotypes, which was carried out in the C57BL/6J background. (B) The affected mutant mouse (aff.) showed hindlimb polydactyly (the additional toe close to the hallux pointed by an arrow), which was not shown in wildtype (+/+). Scale bar, 0.5 cm. (C) The pedigree of the mutant family with the polydactyly phenotype inherited in a dominant manner. Black, affected; white, unaffected.

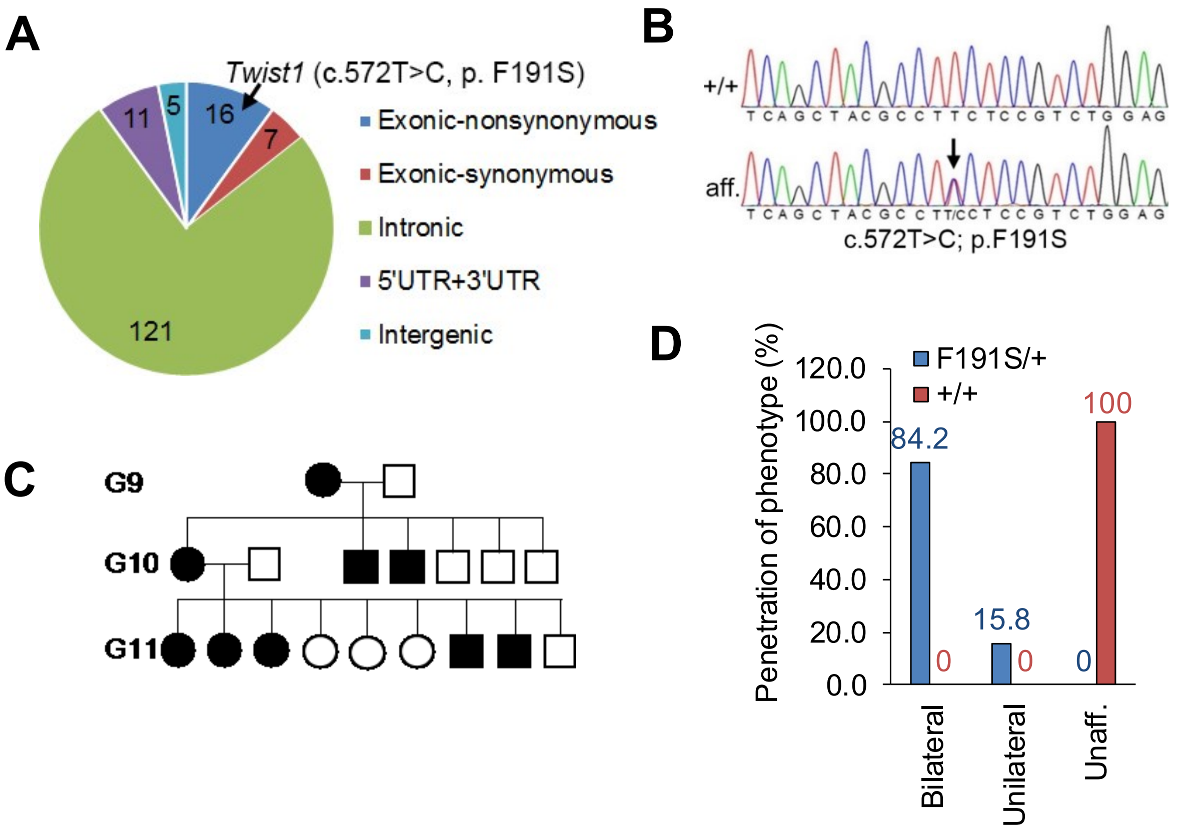

Supplementary Fig. 2. Identification of Twist1-F191S responsible for the hindlimb polydactyly. (A) 160 ENU-induced mutations were identified in a G2 affected mouse by exome capture and DNA sequencing. Among these 160 mutations, 16 are exonic nonsynonymous mutations. Twist1 was identified as the candidate mutant gene responsible for the hindlimb polydactyly (details seen in Table 1). (B) DNA chromatograms showed a non-synonymous mutation (indicated by an arrow) in Twist1 (c.572T>C, p.F191S, NP_035788.1) in affected (aff.) but not wildtype control (+/+) genomic DNA. (C) The G10 and G11 pedigree of the mutant family with the polydactyly phenotype inherited in a dominant manner. 100% heterozygous progenies (F191S/+) presented the hindlimb polydactyly after backcrossing to C57BL/6J for at least ten generations. Black (F191S/+), affected; white (+/+), unaffected. (D) The percentage of unilateral (15.8%) and bilateral (84.2%) hindlimb polydactyly in G10 and G11 offspring. F191S/+, n=19; +/+, n=20.

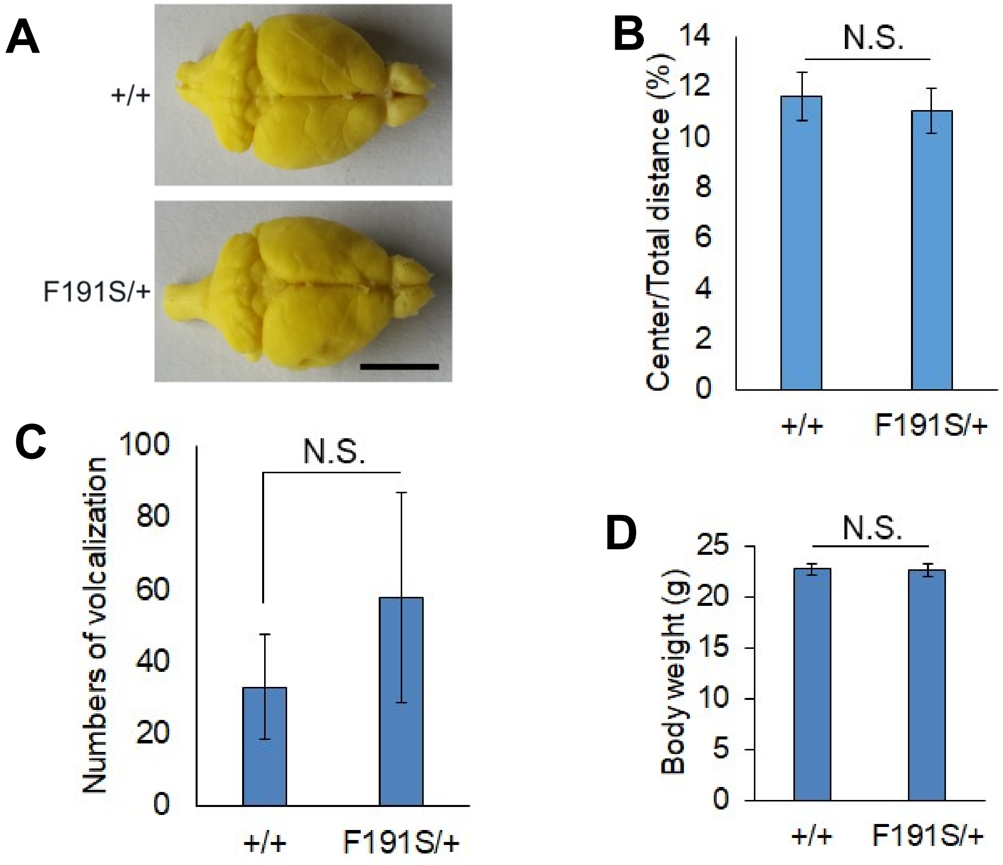

Supplementary Fig. 3. Behaviors, brain size, and body weight were measured in the Twist1-F191S mutant mice. (A) The brain size of indicated genotypes. The brains were fixed with Bouin's solution. Age, 3.5 months. (B) In open field assay, the distance traveled in the center of the field was divided by the total travel distance, which was used to evaluate the anxiety-like behavior. +/+, n=11; F191S/+, n=11. Age, 2 months, male. (C) The times of ultrasonic vocalization (USV) in 5-minute interval after maternal deprivation. Age, P7; +/+, n=26; F191S/+, n=13. (D) Body weight measurement in the wildtype (n=6) and Twist1-F191S mutant (n=6) groups. Age, 2.5 months. The values are presented as mean $\pm$ SEM. N.S., no statistical significance (t-test, SPSS).

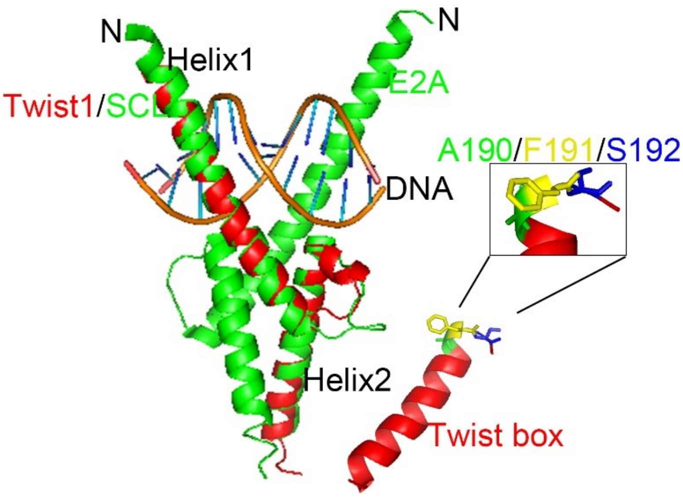

Supplementary Fig. 4. Three dimensional structure prediction for E2A-Twist1-DNA complex. The SCL/E2A structure has been solved previously (RCSB Protein Data Bank, accession numbers of 2YPB). The Twist1 DNA binding domain (red) adopted similar conformation to SCL (green) predicted by SWISS-MODEL. The Twist Box domain forms an α-helical structure and connects to Twist1 DNA binding domain through a flexible loop. The amino acids (Ala190, Phe191, and Ser192) were labeled in green, yellow, and blue, respectively. The whole structure was arranged by PyMOL program.

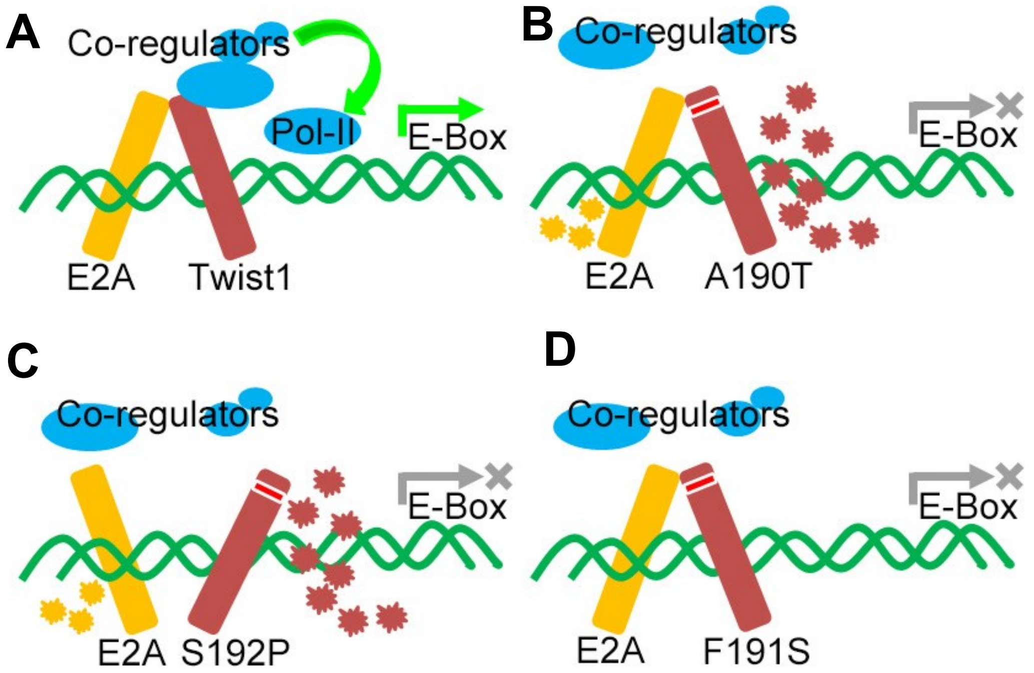

Supplementary Fig. 5. The working model about how the adjacent TA domain mutations affect E2A-dependent Twist1 transcription through different mechanisms. (A) The heterodimer of E2A and Twist1 binds to E-Box DNA and recruits RNA polymerase II (Pol-II) and transcriptional co-regulators to transcribe target genes. (B) Twist1-A190T impairs TA domain C-terminal α-helical structure, leading to proteasome-mediated Twist1 degradation. However, Twist1-A190T still interacts with E2A. (C) Substitution of S192 to proline, a rigid amino acid, severely changes local structure of TA domain, leading to degradation of Twist1 protein and impairment of Twist1/E2A dimerization. (D) Unlike Twist1-A190T and Twist1-S192P, our mutant Twist1 (p.F191S) forms heterodimer with E2A. The hydrophobic side chain of F191 protrudes at the C-terminal α-helical edge, which may serve as interface for protein/protein interaction. Therefore, we hypothesize that our mutation may affect recruitment of co-regulator(s) to the heterodimer, which compromises the transcriptional activity in a dominant-negative manner.

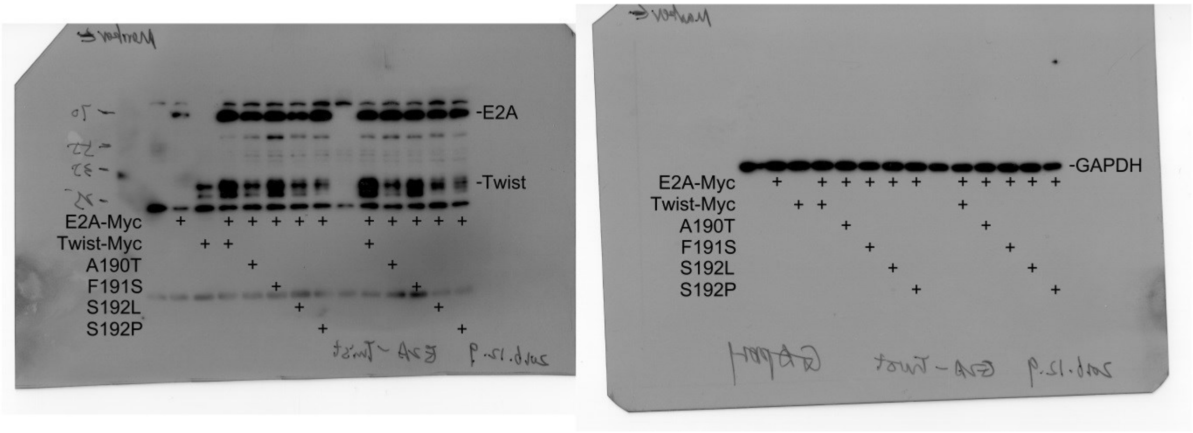

Supplementary Fig. 6. Original uncropped western blot images adopted in Figure 4A.
