# Supplementary material for: An ENU-induced mutation in Twist1 transactivation domain causes hindlimb polydactyly with complete penetrance and dominant-negatively impairs E2A-dependent transcription
Source: Sci Rep. 2020 Feb 12;10:2501. doi: 10.1038/s41598-020-59455-9 (PMC7016005; doi:10.1038/s41598-020-59455-9)
Supplement: Supplementary file 2 — Supplementary Table 1. [file 41598_2020_59455_MOESM2_ESM.pdf]

Supplementary Table 1. The missense and nonsense *TWIST1* mutations

Case	cDNA	Protein	Missense/nonsense	Reported phenotype
1	c.7C>T	p.Q3*	Nonsense	Saethre-Chotzen syndrome
2	c.61G>T	p.E21*	Nonsense	Saethre-Chotzen syndrome
3	c.79C>T	p.Q27*	Nonsense	Saethre-Chotzen syndrome
4	c.82C>T	p.Q28*	Nonsense	Saethre-Chotzen syndrome
6	c.106G>T	p.G36*	Nonsense	Saethre-Chotzen syndrome
8	c.160G>T	p.G54*	Nonsense	Saethre-Chotzen syndrome
9	c.181G>T	p.G61*	Nonsense	Saethre-Chotzen syndrome
10	c.193G>T	p.E65*	Nonsense	Saethre-Chotzen syndrome
11	c.211C>T	p.Q71*	Nonsense	Saethre-Chotzen syndrome
14	c.309C>A	p.Y103*	Nonsense	Saethre-Chotzen syndrome
15	c.309C>G	p.Y103*	Nonsense	Saethre-Chotzen syndrome
16	c.310G>T	p.E104*	Nonsense	Saethre-Chotzen syndrome
17	c.319C>T	p.Q107*	Nonsense	Saethre-Chotzen syndrome
18	c.325C>T	p.Q109*	Nonsense	Saethre-Chotzen syndrome
30	c.355C>T	p.Q119*	Nonsense	Saethre-Chotzen syndrome
34	c.364C>T	p.Q122*	Nonsense	Saethre-Chotzen syndrome
35	c.368C>A	p.S123*	Nonsense	Saethre-Chotzen syndrome
38	c.376G>T	p.E126*	Nonsense	Saethre-Chotzen syndrome
46	c.397A>T	p.K133*	Nonsense	Saethre-Chotzen syndrome
74	c.465C>A	p.Y155*	Nonsense	Saethre-Chotzen syndrome
82	c.480C>G	p.Y160*	Nonsense	Saethre-Chotzen syndrome
83	c.481C>T	p.Q161*	Nonsense	Saethre-Chotzen syndrome
85	c.490C>T	p.Q164*	Nonsense	Saethre-Chotzen syndrome
87	c.541G>T	p.E181*	Nonsense	Baller-Gerold syndrome
91	c.570G>A	p.W190*	Nonsense	Saethre-Chotzen syndrome
5	c.94G>A	p.G32S	Missense	Saethre-Chotzen syndrome
7	c.115C>G	p.R39G	Missense	Saethre-Chotzen syndrome
12	c.247G>A	p.G83S	Missense	Ventricular septal defect
13	c.283A>G	p.S95G	Missense	Ventricular septal defect
19	c.340A>G	p.N114D	Missense	Saethre-Chotzen syndrome
20	c.341A>G	p.N114S	Missense	Saethre-Chotzen syndrome
21	c.346C>G	p.R116G	Missense	Saethre-Chotzen syndrome
22	c.346C>T	p.R116	Missense	Saethre-Chotzen syndrome
23	c.350A>T	p.E117V	Missense	Craniosynostosis, hypertelorism, facial dysmorphology and bilateral undecended testes
24	c.352C>T	p.R118C	Missense	Saethre-Chotzen syndrome
25	c.352C>G	p.R118G	Missense	Saethre-Chotzen syndrome
26	c.353G>A	p.R118H	Missense	Saethre-Chotzen syndrome
27	c.353G>C	p.R118P	Missense	Saethre-Chotzen syndrome
28	c.352C>A	p.R118S	Missense	Saethre-Chotzen syndrome
29	c.356A>C	p.Q119P	Missense	Saethre-Chotzen syndrome
31	c.358C>T	p.R120C	Missense	Saethre-Chotzen syndrome
32	c.359G>C	p.R120P	Missense	Saethre-Chotzen syndrome
33	c.362C>T	p.T121I	Missense	Craniosynostosis
36	c.368C>G	p.S123	Missense	Saethre-Chotzen syndrome
37	c.371T>C	p.L124P	Missense	Saethre-Chotzen syndrome
39	c.380C>A	p.A127E	Missense	Saethre-Chotzen syndrome
40	c.379G>C	p.A127P	Missense	Saethre-Chotzen syndrome
41	c.380C>T	p.A127V	Missense	Saethre-Chotzen syndrome
42	c.392T>C	p.L131P	Missense	Saethre-Chotzen syndrome

43	c.395G>T	p.R132L	Missense	Saethre-Chotzen syndrome
44	c.395G>C	p.R132P	Missense	Saethre-Chotzen syndrome
45	c.394C>T	p.R132	Missense	Saethre-Chotzen syndrome
47	c.402C>G	p.I134M	Missense	Saethre-Chotzen syndrome
48	c.405C>G	p.I135M	Missense	Saethre-Chotzen syndrome
49	c.406C>G	p.P136A	Missense	Saethre-Chotzen syndrome
50	c.407C>A	p.P136H	Missense	Saethre-Chotzen syndrome
51	c.407C>T	p.P136L	Missense	Saethre-Chotzen syndrome
52	c.406C>T	p.P136S	Missense	Saethre-Chotzen syndrome
53	c.409A>C	p.T137P	Missense	Saethre-Chotzen syndrome
54	c.416C>A	p.P139H	Missense	Saethre-Chotzen syndrome
55	c.416C>T	p.P139L	Missense	Saethre-Chotzen syndrome
56	c.415C>T	p.P139S	Missense	Saethre-Chotzen syndrome
57	c.415C>A	p.P139T	Missense	Saethre-Chotzen syndrome
58	c.418T>C	p.S140P	Missense	Saethre-Chotzen syndrome
59	c.421G>A	p.D141N	Missense	Saethre-Chotzen syndrome
60	c.422A>G	p.D141G	Missense	Saethre-Chotzen syndrome
61	c.421G>C	p.D141H	Missense	Saethre-Chotzen syndrome
62	c.421G>T	p.D141Y	Missense	Saethre-Chotzen syndrome
63	c.430A>C	p.S144R	Missense	Saethre-Chotzen syndrome
64	c.435G>C	p.K145N	Missense	Saethre-Chotzen syndrome
65	c.433A>G	p.K145E	Missense	Saethre-Chotzen syndrome
66	c.442A>G	p.T148A	Missense	Saethre-Chotzen syndrome
67	c.443C>A	p.T148N	Missense	Saethre-Chotzen syndrome
68	c.443C>T	p.T148I	Missense	Saethre-Chotzen syndrome
69	c.443C>G	p.T148S	Missense	Saethre-Chotzen syndrome
70	c.445C>T	p.L149F	Missense	Saethre-Chotzen syndrome
71	c.454G>C	p.A152P	Missense	Saethre-Chotzen syndrome
72	c.455C>T	p.A152V	Missense	Saethre-Chotzen syndrome
73	c.460A>G	p.R154G	Missense	Saethre-Chotzen syndrome
75	c.467T>A	p.I156N	Missense	Saethre-Chotzen syndrome
76	c.466A>G	p.I156V	Missense	Baller-Gerold syndrome
77	c.470A>C	p.D157A	Missense	Saethre-Chotzen syndrome
78	c.470A>T	p.D157V	Missense	Saethre-Chotzen syndrome
79	c.472T>C	p.F158L	Missense	Saethre-Chotzen syndrome
80	c.475C>T	p.L159F	Missense	Saethre-Chotzen syndrome
81	c.479A>G	p.Y160C	Missense	Craniosynostosis, nonsyndromic
84	c.487C>T	p.L163F	Missense	Saethre-Chotzen syndrome
86	c.528C>G	p.S176R	Missense	Craniosynostosis
88	c.556G>A	p.A186T	Missense	Craniosynostosis
89	c.561C>G	p.F187L	Missense	Saethre-Chotzen syndrome
90	c.563C>T	p.S188L	Missense	Craniosynostosis
92	c.572G>T	p.R191	Missense	Saethre-Chotzen syndrome
93	c.602C>A	p.S201Y	Missense	Craniosynostosis

Note: The disease-associated mutations were annotated by HGMD amd OMI[illegible]
amino acid positioning.

s.

Reference
Elanko (2001) Hum Mutat 18, 535;Piard (2015) Clin Genet 87:
Kress (2006) Eur J Hum Genet 14, 39
Roscioli (2013) Am J Med Genet C Semin Med Genet 163, 259
Gripp (2000) Hum Mutat 15, 150
Elanko (2001) Hum Mutat 18, 535
Aref-Eshghi (2018) Am J Hum Genet 102, 156
Rose (1997) Hum Mol Genet 6, 1369
Rose (1997) Hum Mol Genet 6, 1369
Cai (2003) Clin Genet 64, 79
El Ghouzzi (1997) Nat Genet 15, 42
Paznekas (1998) Am J Hum Genet 62, 1370
Paznekas (1998) Am J Hum Genet 62, 1370
James (2009) Genes Chromosomes Cancer 48, 533
Yousfi (2002) Hum Mol Genet 11, 359
Kress (2006) Eur J Hum Genet 14, 39
Paznekas (1998) Am J Hum Genet 62, 1370
El Ghouzzi (1997) Nat Genet 15, 42
El Ghouzzi (1997) Nat Genet 15, 42
Ko (2012) Plast Reconstr Surg 129, 814e
Elanko (2001) Hum Mutat 18, 535
Gripp (2000) Hum Mutat 15, 150
Paznekas (1998) Am J Hum Genet 62, 1370
Gripp (2000) Hum Mutat 15, 150
Gripp (1999) Am J Med Genet 82, 170
Seifert (2006) Cancer Genet Cytogenet 171, 76
Foo (2009) Plast Reconstr Surg 124, 2085
Funato (2005) Hum Mutat 25, 550
Deng (2015) Pediatr Cardiol epub, epub
Deng (2015) Pediatr Cardiol epub, epub
Woods (2009) Plast Reconstr Surg 123, 1801
de Heer (2005) Plast Reconstr Surg 115, 1894
de Heer (2005) Plast Reconstr Surg 115, 1894
Paznekas (1998) Am J Hum Genet 62, 1370
Miller (2017) J Med Genet 54, 260
Yousfi (2002) Hum Mol Genet 11, 359;El Ghouzzi (2001) FEBS Lett 492: 112 ;Maia (2012) BMC Bioinformatics 13: 184
Aref-Eshghi (2018) Am J Hum Genet 102, 156
Rose (1997) Hum Mol Genet 6, 1369
Kress (2006) Eur J Hum Genet 14, 39
Foo (2009) Plast Reconstr Surg 124, 2085;Berg (2013) Genet Med 15: 36
Howard (1997) Nat Genet 15, 36
de Heer (2005) Plast Reconstr Surg 115, 1894
Gripp (2000) Hum Mutat 15, 150
Wilkie (2006) Am J Med Genet A 140A, 2631;Roscioli (2013) Am J Med Genet C Semin Med Genet 163: 259;Wilkie (2007) Am J Med Genet A 143A: 1941
Johnson (1998) Am J Hum Genet 63, 1282
Roscioli (2013) Am J Med Genet C Semin Med Genet 163, 259
Kress (2006) Eur J Hum Genet 14, 39
Seifert (2006) Cancer Genet Cytogenet 171, 76
Gripp (2000) Hum Mutat 15, 150
El Ghouzzi (1997) Nat Genet 15, 42

James (2009) Genes Chromosomes Cancer 48, 533
Paznekas (1998) Am J Hum Genet 62, 1370
Paumard-Hernández (2015) Eur J Hum Genet 23, 907
Rose (1997) Hum Mol Genet 6, 1369
Woods (2009) Plast Reconstr Surg 123, 1801
Meng (2017) JAMA Pediatr 171, e173438
de Heer (2005) Plast Reconstr Surg 115, 1894;Lamônica (2010) Cleft Palate Craniofac J 47: 548
Johnson (1998) Am J Hum Genet 63, 1282
de Heer (2005) Plast Reconstr Surg 115, 1894
Kress (2006) Eur J Hum Genet 14, 39
Elanko (2001) Hum Mutat 18, 535
Chun (2002) Am J Med Genet 110, 136
Paznekas (1998) Am J Hum Genet 62, 1370
Woods (2009) Plast Reconstr Surg 123, 1801
Ko (2012) Plast Reconstr Surg 129, 814e
Tahiri (2015) J Craniofac Surg 26, 1564
Rose (1997) Hum Mol Genet 6, 1369
James (2009) Genes Chromosomes Cancer 48, 533
Paznekas (1998) Am J Hum Genet 62, 1370
El Ghouzzi (1999) Eur J Hum Genet 7, 27;El Ghouzzi (2001) FEBS Lett 492: 112;Maia (2012) BMC Bioinformatics 13: 184
Rose (1997) Hum Mol Genet 6, 1369
Paznekas (1998) Am J Hum Genet 62, 1370;El Ghouzzi (2001) FEBS Lett 492: 112;Maia (2012) BMC Bioinformatics 13: 184
Gripp (2000) Hum Mutat 15, 150
Paznekas (1998) Am J Hum Genet 62, 1370
Kress (2006) Eur J Hum Genet 14, 39
Kress (2006) Eur J Hum Genet 14, 39
Paznekas (1998) Am J Hum Genet 62, 1370;Piard (2012) Eur J Med Genet 55: 719
Elanko (2001) Hum Mutat 18, 535
Paznekas (1998) Am J Hum Genet 62, 1370
Rose (1997) Hum Mol Genet 6, 1369
Woods (2009) Plast Reconstr Surg 123, 1801
Seto (2001) Am J Med Genet 104, 323
de Heer (2005) Plast Reconstr Surg 115, 1894
Kress (2006) Eur J Hum Genet 14, 39
Elanko (2001) Hum Mutat 18, 535
Paznekas (1998) Am J Hum Genet 62, 1370
Timberlake (2017) Proc Natl Acad Sci U S A 114, E7341
Paumard-Hernández (2015) Eur J Hum Genet 23, 907;Xu (2018) Gene 641: 144
Bessenyei (2015) Am J Med Genet A 167A, 2985;Xu (2018) Gene 641: 144
Seto (2007) Am J Med Genet A 143A, 678
Kress (2006) Eur J Hum Genet 14, 39
Seto (2007) Am J Med Genet A 143A, 678
Pena (2010) Cleft Palate Craniofac J 47, 318
Kress (2006) Eur J Hum Genet 14, 39;Seto (2007) Am J Med Genet A 143: 678

M. The transcript (ENST00000242261) of *TWIST1* was used for
