# Supplementary material for: An ENU-induced mutation in Twist1 transactivation domain causes hindlimb polydactyly with complete penetrance and dominant-negatively impairs E2A-dependent transcription
Source: Sci Rep. 2020 Feb 12;10:2501. doi: 10.1038/s41598-020-59455-9 (PMC7016005; doi:10.1038/s41598-020-59455-9)
Supplement: Supplementary file 3 — Supplementary Table 2. [file 41598_2020_59455_MOESM3_ESM.pdf]

Supplementary Table 2. The small indels of *TWIST1* .

Case	cDNA	Category	Reported phenotype
1	c.31_32delTC	small deletion	Craniosynostosis
2	c.108delA	small deletion	Saethre-Chotzen syndrome
3	c.132_142del11	small deletion	Saethre-Chotzen syndrome
4	c.127_137del11	small deletion	Saethre-Chotzen syndrome
5	c.149delC	small deletion	Saethre-Chotzen syndrome
6	c.156delC	small deletion	Saethre-Chotzen syndrome
7	c.176delG	small deletion	Craniosynostosis
8	c.200delG	small deletion	Saethre-Chotzen syndrome
9	c.220delC	small deletion	Saethre-Chotzen syndrome
10	c.230delA	small deletion	Saethre-Chotzen syndrome
11	c.246delC	small deletion	Saethre-Chotzen syndrome
12	c.263_275del13	small deletion	Saethre-Chotzen syndrome
13	c.263delG	small deletion	Saethre-Chotzen syndrome
14	c.309delC	small deletion	Saethre-Chotzen syndrome
15	c.326_342del17	small deletion	Saethre-Chotzen syndrome
16	c.329_333delGGGTC	small deletion	Craniosynostosis
17	c.331delG	small deletion	Saethre-Chotzen syndrome
18	c.337delG	small deletion	Saethre-Chotzen syndrome
19	c.348_364del17	small deletion	Saethre-Chotzen syndrome
20	c.353_355delGCC	small deletion	Saethre-Chotzen syndrome
21	c.353_360delGCCAGCGC	small deletion	Saethre-Chotzen syndrome
22	c.355delC	small deletion	Saethre-Chotzen syndrome
23	c.428delT	small deletion	Saethre-Chotzen syndrome
24	c.465_469delCATCG	small deletion	Saethre-Chotzen syndrome
25	c.481delC	small deletion	Saethre-Chotzen syndrome
26	c.482_488delAGGTCCT	small deletion	Saethre-Chotzen syndrome
27	c.485_488delTCCT	small deletion	Craniosynostosis
28	c.487delC	small deletion	Saethre-Chotzen syndrome
29	c.190_200dup11	small insertion	Saethre-Chotzen syndrome
30	c.276_277ins10	small insertion	Saethre-Chotzen syndrome
31	c.260_274dup15	small insertion	Craniosynostosis
32	c.272_281dup10	small insertion	Saethre-Chotzen syndrome
33	c.308dupA	small insertion	Saethre-Chotzen syndrome
34	c.338_339dupCC	small insertion	Saethre-Chotzen syndrome
35	c.379_381dupGCG	small insertion	Saethre-Chotzen syndrome
36	c.384dupC	small insertion	Saethre-Chotzen syndrome
37	c.408dupC	small insertion	Saethre-Chotzen syndrome
38	c.434dupA	small insertion	Craniosynostosis, syndromic
39	c.438dupT	small insertion	Saethre-Chotzen syndrome
40	c.460dupA	small insertion	Robinow-Sorauf syndrome
41	c.477_478dupCT	small insertion	Craniosynostosis
42	c.81_82delGCinsTT	small indels	Saethre-Chotzen syndrome
43	c.230_232delAGTinsGC	small indels	Saethre-Chotzen syndrome
44	c.283delAinsCG	small indels	Saethre-Chotzen syndrome
45	c.356_357delAGinsCC	small indels	Saethre-Chotzen syndrome
46	c.380_399delins20	small indels	Saethre-Chotzen syndrome

Note: The disease-associated mutations were annotated by HGMD amd OMIM. Th
amino acid positioning.

Reference
Roscioli (2013) Am J Med Genet C Semin Med Genet 163,
Kress (2006) Eur J Hum Genet 14, 39
Cai (2003) Hum Genet 114, 68
Boeck (2001) Am J Med Genet 104, 53
Aref-Eshghi (2018) Am J Hum Genet 102, 156
Aref-Eshghi (2018) Am J Hum Genet 102, 156
Wilkie (2006) Am J Med Genet A 140A, 2631；Wilkie (2007) Am J Med Genet A 143A: 1941
Paumard-Hernández (2015) Eur J Hum Genet 23, 907
Ko (2012) Plast Reconstr Surg 129, 814e
James (2009) Genes Chromosomes Cancer 48, 533
Foo (2009) Plast Reconstr Surg 124, 2085
Aref-Eshghi (2018) Am J Hum Genet 102, 156
Gripp (2000) Hum Mutat 15, 150
Cai (2003) Hum Genet 114, 68
de Heer (2005) Plast Reconstr Surg 115, 1894
Wilkie (2006) Am J Med Genet A 140A, 2631；Wilkie (2007) Am J Med Genet A 143A: 1941
Woods (2009) Plast Reconstr Surg 123, 1801
Kress (2006) Eur J Hum Genet 14, 39
Chun (2002) Am J Med Genet 110, 136
Paznekas (1998) Am J Hum Genet 62, 1370
El Ghouzzi (1999) Eur J Hum Genet 7, 27
Elanko (2001) Hum Mutat 18, 535
Kress (2006) Eur J Hum Genet 14, 39
El Ghouzzi (1999) Eur J Hum Genet 7, 27
Elanko (2001) Hum Mutat 18, 535
Cai (2003) Hum Genet 114, 68
Elanko (2001) Hum Mutat 18, 535
El Ghouzzi (1999) Eur J Hum Genet 7, 27
James (2009) Genes Chromosomes Cancer 48, 533
Gripp (2000) Hum Mutat 15, 150
Elanko (2001) Hum Mutat 18, 535
Foo (2009) Plast Reconstr Surg 124, 2085
Howard (1997) Nat Genet 15, 36
Foo (2009) Plast Reconstr Surg 124, 2085
Elanko (2001) Hum Mutat 18, 535
Paznekas (1998) Am J Hum Genet 62, 1370
Roscioli (2013) Am J Med Genet C Semin Med Genet 163,
Xu (2018) Gene 641, 144
Roscioli (2013) Am J Med Genet C Semin Med Genet 163,
Kunz (1999) J Med Genet 36, 650
Wilkie (2006) Am J Med Genet A 140A, 2631；Wilkie (2007) Am J Med Genet A 143A: 1941
James (2009) Genes Chromosomes Cancer 48, 533
Rose (1997) Hum Mol Genet 6, 1369；Johnson (1998) Am J Hum Genet 63: 1282
Elanko (2001) Hum Mutat 18, 535
Altiner (2017) Clin Dysmorphol 26, 175
Kress (2006) Eur J Hum Genet 14, 39

ne transcript (ENST00000242261) of *TWIST1* was used for
