# Supplementary material for: An ENU-induced mutation in Twist1 transactivation domain causes hindlimb polydactyly with complete penetrance and dominant-negatively impairs E2A-dependent transcription
Source: Sci Rep. 2020 Feb 12;10:2501. doi: 10.1038/s41598-020-59455-9 (PMC7016005; doi:10.1038/s41598-020-59455-9)
Supplement: Supplementary file 4 — Supplementary Table 3. [file 41598_2020_59455_MOESM4_ESM.pdf]

Supplementary Table 3. The large indels of *TWIST1*.

Case	DNA level	Description	Category
1	gDNA	<1969kb incl entire gene, FERD3L & 3 others	Gross deletion
2	gDNA	>1874bp incl entire gene	Gross deletion
3	gDNA	>797 bp	Gross deletion
4	gDNA	~13Mb incl entire gene, HOXA13 & 72 others	Gross deletion
5	gDNA	1 Mb, 240 kb from gene	Gross deletion
6	gDNA	11.4Mb incl entire gene & 53 others	Gross deletion
7	gDNA	11.7 mb incl. entire gene + FERD3L	Gross deletion
8	gDNA	12 Mb incl. entire gene	Gross deletion
9	gDNA	14.7 Mb incl entire gene + 40 others	Gross deletion
10	cDNA	21 bp nt 394	Gross deletion
11	gDNA	23 bp nt. 433 cd. 145	Gross deletion
12	cDNA	26 bp nt 120-145	Gross deletion
13	gDNA	4.37 Mb incl. entire gene	Gross deletion
14	gDNA	5.5 Mb - 7.8 Mb	Gross deletion
15	gDNA	5.5Mb incl entire gene & 22 others	Gross deletion
16	gDNA	510 kb - 16 Mb	Gross deletion
17	gDNA	526 kb incl. entire gene + FERD3L	Gross deletion
18	gDNA	600 kb - 8.7 Mb	Gross deletion
19	gDNA	797 bp - 14.4 Mb	Gross deletion
20	gDNA	797 bp - 510 kb	Gross deletion
21	gDNA	8.72 Mb incl. entire gene	Gross deletion
22	gDNA	9.2 mb incl. entire gene + FERD3L	Gross deletion
23	gDNA	Del (7)(p15.1p21.3)	Gross deletion
24	gDNA	Del (7)(p21p21)	Gross deletion
25	gDNA	ex. 1	Gross deletion
26	gDNA	ex. 1-2	Gross deletion
27	gDNA	incl. entire gene	Gross deletion
28	cDNA	Duplication,21 bp c.396_416	Gross insertion
29	cDNA	Duplication,21 bp c.397_417	Gross insertion
30	cDNA	Insertion, 21 bp nt 276	Gross insertion
31	gDNA	Duplication,21 bp nt 385-405	Gross insertion
32	gDNA	Insertion, 21 bp nt 406	Gross insertion
33	cDNA	Duplication,21 bp nt. 405	Gross insertion
34	cDNA	Duplication,21 bp nt. 416	Gross insertion
35	cDNA	Duplication,21 bp nt. 417	Gross insertion
36	cDNA	Duplication,21 bp nt. 418	Gross insertion
37	cDNA	Duplication,21 bp nt. 419	Gross insertion
38	cDNA	Duplication,21 bp nt. 421	Gross insertion
39	gDNA	Duplication,22 bp c.416_437	Gross insertion
40	cDNA	Insertion, 25 bp nt 423	Gross insertion
41	gDNA	Duplication,35 bp c.243_277	Gross insertion
42	gDNA	Duplication,c.397_418dup	Gross insertion
43	gDNA	Duplication,C.401_421dup	Gross insertion
44	gDNA	Ala127Thr, Lys133Ile	Complex rearrangements
45	gDNA	Balanced translocation t(7;12)(p21.2;p12.3), 42kb 3' of gene	Complex rearrangements
46	gDNA	Del 2924 bp, ins GT nt 10962	Complex rearrangements

47	gDNA	Inversion (7)(p21.3q34), 260 kb 3' of the	Complex rearrangements
48	gDNA	t(2;7)(p24;p21),ins(7)(p21.3q21.3q22).ish del(7)(p21.3)	Complex rearrangements
49	gDNA	t(7;18)(p15.2;q12.1), interstitial del. of chr. 7 material	Complex rearrangements
50	gDNA	Translocation (2;7)	Complex rearrangements
51	gDNA	Translocation t(1;4;7)(q42.13;q31.3;p22)	Complex rearrangements
52	gDNA	Translocation t(2;7)(p23;p22)	Complex rearrangements
53	gDNA	Translocation t(6;7)(q16.2;p15.3)	Complex rearrangements
54	gDNA	Translocation t(7;8)(p21;q13)	Complex rearrangements

Note: The disease-associated mutations were annotated by HGMD amd OMIM.

Reported phenotype	Reference
Saethre-Chotzen syndrome & hyper IgE syndrome	Zechi-Ceide (2012) Am J Med Genet A 158A, 1680
Saethre-Chotzen syndrome	Aradhya (2012) Genet Med 14, 594
Saethre-Chotzen syndrome	Cai (2003) Hum Genet 114, 68
Saethre-Chotzen syndrome & hand-foot-uterus syndrome	Fryssira (2011) Mol Syndromol 2, 45
Saethre-Chotzen syndrome	Tahiri (2015) J Craniofac Surg 26, 1564
Saethre-Chotzen syndrome	Spaggiari (2012) Eur J Med Genet 55, 498
Microcephaly, facial dysmorphism and short stature	Busche (2011) Eur J Med Genet 54, 256
Saethre-Chotzen syndrome	Tahiri (2015) J Craniofac Surg 26, 1564
Dysmorphic features	Schinagl (2017) J Med Case Rep 11, 226
Saethre-Chotzen syndrome	Aref-Eshghi (2018) Am J Hum Genet 102, 156
Saethre-Chotzen syndrome	Howard (1997) Nat Genet 15, 36
Saethre-Chotzen syndrome	Aref-Eshghi (2018) Am J Hum Genet 102, 156
Saethre-Chotzen syndrome	Cho (2013) Childs Nerv Syst 29, 2101
Saethre-Chotzen syndrome	Cai (2003) Hum Genet 114, 68
Saethre-Chotzen-like syndrome, intellectual disability	Shimada (2013) Am J Med Genet A 161, 2078
Saethre-Chotzen syndrome	Cai (2003) Hum Genet 114, 68
Microcephaly, facial dysmorphism and short stature	Busche (2011) Eur J Med Genet 54, 256
Saethre-Chotzen syndrome	Cai (2003) Hum Genet 114, 68
Saethre-Chotzen syndrome	Cai (2003) Hum Genet 114, 68
Saethre-Chotzen syndrome	Cai (2003) Hum Genet 114, 68
Saethre-Chotzen syndrome	Tahiri (2015) J Craniofac Surg 26, 1564
Microcephaly, facial dysmorphism and short stature	Busche (2011) Eur J Med Genet 54, 256
Saethre-Chotzen syndrome	Woods (2009) Plast Reconstr Surg 123, 1801
Saethre-Chotzen syndrome	Woods (2009) Plast Reconstr Surg 123, 1801;Wilkie (2006) Am J Med Genet A 140: 2631;Wilkie (2007) Am J Med Genet A 143A:
Saethre-Chotzen syndrome	Roscioli (2013) Am J Med Genet C Semin Med Genet 163, 259
Saethre-Chotzen syndrome	Paumard-Hernández (2015) Eur J Hum Genet 23, 907
Saethre-Chotzen syndrome	Johnson (1998) Am J Hum Genet 63, 1282
Craniosynostosis	Wilkie (2006) Am J Med Genet A 140A, 2631;Tahiri (2015) J Craniofac Surg 26: 1564;Wilkie (2007) Am J Med Genet A 143A:
Craniosynostosis	Wilkie (2006) Am J Med Genet A 140A, 2631;Wilkie (2007) Am J Med Genet A 143A: 1941
Saethre-Chotzen syndrome	Gripp (2000) Hum Mutat 15, 150;Elanko (2001) Hum Mutat 18:
Saethre-Chotzen syndrome	Aref-Eshghi (2018) Am J Hum Genet 102, 156
Saethre-Chotzen syndrome	Aref-Eshghi (2018) Am J Hum Genet 102, 156
Saethre-Chotzen syndrome	Howard (1997) Nat Genet 15, 36
Saethre-Chotzen syndrome	Howard (1997) Nat Genet 15, 36
Saethre-Chotzen syndrome	de Heer (2005) Plast Reconstr Surg 115, 1894
Saethre-Chotzen syndrome	Rose (1997) Hum Mol Genet 6, 1369
Saethre-Chotzen syndrome	Rose (1997) Hum Mol Genet 6, 1369
Saethre-Chotzen syndrome	Rose (1997) Hum Mol Genet 6, 1369
Saethre-Chotzen syndrome	Foo (2009) Plast Reconstr Surg 124, 2085
Saethre-Chotzen syndrome	El Ghouzzi (1999) Eur J Hum Genet 7, 27
Saethre-Chotzen syndrome	Foo (2009) Plast Reconstr Surg 124, 2085
Saethre-Chotzen syndrome	Roscioli (2013) Am J Med Genet C Semin Med Genet 163, 259
Saethre-Chotzen syndrome	Roscioli (2013) Am J Med Genet C Semin Med Genet 163, 259
Saethre-Chotzen syndrome	Kress (2006) Eur J Hum Genet 14, 39
Saethre-Chotzen-like syndrome	De Marco (2011) Eur J Med Genet 54, e478
Saethre-Chotzen syndrome	Johnson (1998) Am J Hum Genet 63, 1282

Saethre-Chotzen syndrome	Cai (2003) Hum Genet 114, 68
Saethre-Chotzen syndrome	Schluth-Bolard (2008) Eur J Med Genet 51, 156
Craniosynostosis	Shetty (2007) Clin Dysmorphol 16, 253
Saethre-Chotzen syndrome	Foo (2009) Plast Reconstr Surg 124, 2085
Saethre-Chotzen syndrome	Massalska (2014) Ginekol Pol 85, 541
Saethre-Chotzen syndrome	Lewanda (1994) Am J Hum Genet 55, 1195
Saethre-Chotzen syndrome	Krebs (1997) Hum Mol Genet 6, 1079
Saethre-Chotzen syndrome	Johnson (1998) Am J Hum Genet 63, 1282
