# Supplementary material for: An ENU-induced mutation in Twist1 transactivation domain causes hindlimb polydactyly with complete penetrance and dominant-negatively impairs E2A-dependent transcription
Source: Sci Rep. 2020 Feb 12;10:2501. doi: 10.1038/s41598-020-59455-9 (PMC7016005; doi:10.1038/s41598-020-59455-9)
Supplement: Supplementary file 5 — Supplementary Table 4. [file 41598_2020_59455_MOESM5_ESM.pdf]

Supplementary Table 4. Number of P10 pups carry craniofacial abnormalities.

Craniofacial abnormalities	+/+	F191S/+
Pre-fusion of sagittal suture	0	6
Pre-fusion of coronal suture	0	0
Pre-fusion of lambdoid suture	0	0
Total pups we analyzed	6	11

Note: No obvious coronal and lambdoid pre-fusion was detected in the F191S/+ pups we analyzed.
